# Effectiveness of Machine Learning in Detecting Vessels Encapsulating Tumor Clusters in Hepatocellular Carcinoma: Systematic Review and Meta-Analysis

**DOI:** 10.2196/82839

**Published:** 2026-01-14

**Authors:** Huili Shui, Wenyu Wu, Zhenming Xie, Bing Yang, Jia Deng, Dongxin Tang

**Affiliations:** 1 Guizhou University of Traditional Chinese Medicine Guiyang, Guizhou Province China; 2 Clinical Medical Research Center, The First Affiliated Hospital of Guizhou University of Traditional Chinese Medicine Guiyang, Guizhou Province China; 3 Guizhou Traditional Chinese Medicine Oncology Heritage and Technology Innovation Talent Base Guiyang, Guizhou Province China

**Keywords:** carcinoma, hepatocellular carcinoma, hepatocellular, machine learning, radiomics, sensitivity, specificity, vessels encapsulating tumor clusters, VETC

## Abstract

**Background:**

Vessels encapsulating tumor clusters (VETC) are significantly associated with poor prognosis in hepatocellular carcinoma (HCC). However, identifying VETC early remains challenging. Recently, machine learning has shown promise for VETC detection, but their diagnostic accuracy lacks systematic validation.

**Objective:**

This meta-analysis aimed to systematically evaluate the diagnostic accuracy of machine learning models for detecting VETC in patients with HCC.

**Methods:**

The Cochrane Library, Embase, Web of Science, and PubMed were searched up to June 21, 2025. Eligible studies focused on machine learning models for HCC VETC diagnosis. Studies that merely analyzed risk factors or lacked outcome measures were excluded. The Prediction Model Risk of Bias Assessment Tool was used to evaluate the risk of bias. A bivariate mixed-effects model was used for a meta-analysis based on 2×2 diagnostic tables. Subgroup analyses were performed according to modeling variables (nonradiomic vs radiomic features) and model types (traditional machine learning vs deep learning).

**Results:**

This meta-analysis included 31 studies comprising 6755 patients with HCC (2699 VETC-positive). Nineteen studies used machine learning models based on nonradiomic features, and 12 used radiomic features (including deep learning). In the validation set, the nonradiomic model demonstrated a pooled sensitivity of 0.72 (95% CI 0.66-0.78), specificity of 0.74 (95% CI 0.68-0.80), and an area under the summary receiver operating characteristic curve (SROC AUC) of 0.80 (95% CI 0.76-0.83). The radiomic model showed sensitivity of 0.81 (95% CI 0.73-0.87), specificity of 0.73 (95% CI 0.67-0.79), and SROC AUC of 0.84 (95% CI 0.80-0.87). Traditional machine learning achieved sensitivity of 0.84 (95% CI 0.71-0.92), specificity of 0.75 (95% CI 0.67-0.81), and SROC AUC of 0.83 (95% CI 0.80-0.86). Deep learning exhibited sensitivity of 0.77 (95% CI 0.69-0.84), specificity of 0.70 (95% CI 0.59-0.79), and SROC AUC of 0.81 (95% CI 0.77-0.85).

**Conclusions:**

This meta-analysis is the first to quantitatively assess the efficacy of machine learning models in HCC VETC diagnosis, addressing an evidence gap in this field. Unlike previous descriptive reviews, this analysis provides the first quantitative evidence revealing the potential value of machine learning in detecting HCC VETC. The findings provide a foundation for developing and refining subsequent intelligent detection tools. Despite their promising prospects, machine learning models have not yet reached the maturity required for clinical translation, owing to methodological heterogeneity, limited validation, and a high risk of bias. Future research should focus on conducting multicenter, large-sample, standardized, prospective studies to advance clinical translation.

**Trial Registration:**

PROSPERO CRD420251084894; https://www.crd.york.ac.uk/PROSPERO/view/CRD420251084894

## Introduction

Liver cancer is the sixth most frequently diagnosed malignancy and the third leading cause of cancer-related mortality worldwide. In 2022, there were approximately 865,000 new cases of liver cancer and 757,948 related deaths. Hepatocellular carcinoma (HCC) accounts for 75% to 85% of primary liver cancers. Higher incidence and mortality rates are predominantly observed in developing regions, including Mongolia, Cambodia, Laos, Thailand, Vietnam, and Egypt [1]. Consequently, HCC has become a significant global oncological concern. For early-stage HCC, curative interventions such as surgical resection, liver transplantation, and ablation are recommended. For intermediate stages, locoregional therapies are typically used, while systemic treatment is preferred for individuals with a significant intrahepatic tumor burden. Advanced HCC is primarily managed with immune checkpoint inhibitors [2]. While these treatments have prolonged the survival of some patients, others still have a poor prognosis, even after undergoing the same treatment regimen. Patients with HCC have an overall 5-year survival rate of less than 20% [3]. Adding to the clinical challenge, the postoperative recurrence rate for HCC is high, at around 70%, even after curative resection. This persistent risk is a primary factor in unfavorable long-term patient prognosis [4]. Several factors have been associated with poor HCC prognosis, including microvascular invasion [5], the macrotrabecular-massive subtype [6], and the coexpression of Ki-67 and cytokeratin 19 [7]. Thus, identifying the key factors that drive poor prognosis in HCC is crucial.

Recently, there has been an increase in attention directed toward a distinct microvascular pattern known as vessels encapsulating tumor clusters (VETC). First described by Fang et al [8] in 2015, VETC refers to a vascular network surrounding tumor clusters in a spiderweb configuration. This pattern has been characterized as an independent vascular morphology that is distinct from epithelial-mesenchymal transition. It facilitates the release of entire tumor clusters into the bloodstream and enables a noninvasive metastatic mechanism in HCC. Research indicates that the prevalence of VETC correlates with tumor stage and aggressiveness. VETC occurs in approximately 30%-40% of patients undergoing resection, 50%-55% of individuals with postresection recurrence, and up to 76% of patients with unresectable disease receiving liver transplants [9]. A positive VETC status substantially influences the long-term prognosis of patients with HCC [10]. Recent studies show that patients with VETC have significantly shorter overall and disease-free survival than patients without VETC [11]. The presence of VETC has been established as a robust predictor of aggressive HCC behavior [10]. A meta-analysis by Wang et al [12] further confirmed VETC as a significant predictor of overall survival and tumor recurrence, supporting its role as an effective prognostic biomarker. Furthermore, multiparameter prognostic models that incorporate VETC status demonstrate superior predictive capacity for disease-free and overall survival in patients with HCC compared to the conventional tumor-node-metastasis staging system. These models facilitate personalized temporal survival estimation and have the potential to enhance clinical decision-making regarding surveillance management and therapeutic strategies [13,14]. Concurrently, VETC status is valuable in guiding systemic therapy selection and predicting treatment response in HCC. Notably, patients with VETC experience greater survival benefits from therapies including sorafenib [15], lenvatinib [16], and transarterial chemoembolization [17] than their VETC-negative counterparts do. These observations suggest that VETC-based stratified treatment strategies may optimize patient outcomes further, providing an evidence base for clinical decision-making [9]. Therefore, early detection of VETC status is clinically relevant for improving HCC prognosis. Currently, a definitive diagnosis of VETC relies on histopathological examination of biopsy or resected tissue specimens. However, this approach has several limitations. Technical challenges include dependence on tumor size and needle gauge, as well as variability among clinicians and pathologists. Procedural risks encompass hemorrhage, seeding metastasis, sampling error, and uncertainty in tumor characterization [18]. Thus, noninvasive methods for identifying VETC status in HCC are urgently needed to circumvent the limitations of tissue acquisition. Recent advancements in image processing and artificial intelligence have sparked growing interest in clinical oncology in the development of predictive models that integrate computed tomography, magnetic resonance imaging (MRI), and contrast-enhanced ultrasound with machine learning algorithms. This methodology is increasingly being explored for the noninvasive diagnosis of HCC VETC [19-21]. Several studies have explored the potential for directly diagnosing VETC in HCC using images alone [22,23]. Furthermore, machine learning models that incorporate clinical and imaging features have been developed to noninvasively predict VETC status [20,24]. Despite these promising findings, there is a lack of systematic evidence substantiating the efficacy of machine learning–based approaches for VETC detection in HCC. This lack of evidence poses a significant challenge to the development and improvement of artificial intelligence–assisted diagnostic tools. To address this deficiency, this systematic review and meta-analysis were conducted to summarize the performance of machine learning in noninvasively detecting VETC in HCC. The aim is to provide evidence-based support for developing and optimizing future intelligent diagnostic tools.

## Methods

### Study Registration

This meta-analysis was conducted in strict accordance with the PRISMA (Preferred Reporting Items for a Systematic Review and Meta-Analysis; checklist provided in Multimedia Appendix 1) Diagnostic Test Accuracy Studies guidelines [25] and was prospectively registered with the International Prospective Register of Systematic Reviews (CRD420251084894).

### Eligibility Criteria

Textbox 1 presents the eligibility criteria for studies.

**Eligibility criteria.**

**Inclusion criteria**

- Participants diagnosed with hepatocellular carcinoma
- Cohort, case-control, or cross-sectional studies
- Studies that developed machine learning models for the diagnosis vessels encapsulating tumor clusters
- Publications reported in English

**Exclusion criteria**

- Reviews, guidelines, expert opinions, or conference abstracts
- Studies that only performed risk factor analyses without constructing machine learning models
- Studies lacking key metrics for assessing the accuracy of machine learning models
- Studies reporting only univariable predictive performance

### Data Sources and Search Strategy

According to the PRISMA search guidelines, the PubMed, Embase, Cochrane Library, and Web of Science databases were searched up to June 21, 2025. The search combined Medical Subject Headings and free-text terms, with no restrictions on language, country, or publication date. The search strategy was developed independently for this analysis. It was not adapted from existing systematic reviews, nor did it incorporate additional information sources or use search filters. The strategy did not undergo peer review before its execution, and no updates were made to the search following the initial retrieval. Based on the existing literature, we manually examined the reference lists of selected studies and relevant reviews to identify additional articles. Conference proceedings were excluded, and no attempts were made to contact authors for additional information [26]. Details are presented in Table S1 in Multimedia Appendix 2.

### Study Selection

All retrieved articles were imported into EndNote (version 21; Clarivate) to remove duplicates. Two researchers (HS and ZX) screened the titles and abstracts of the articles independently and excluded the irrelevant ones. Subsequently, the full texts of potentially eligible studies were acquired and assessed for final inclusion. The researchers then cross-checked their results. Any discrepancies were resolved through discussion or adjudication by a third researcher (WW).

### Data Extraction

Prior to data extraction, a standardized spreadsheet was developed. The extracted data included the following: first author, number of VETC cases, patient source, total sample size, study design, detection method, number of VETC cases in the training set, total training set size, country, method of validation set generation, model type, publication year, total validation set size, number of VETC cases in the validation set, and modeling variables.

### Risk of Bias

The Prediction Model Risk of Bias Assessment Tool was applied to evaluate the risk of bias across four domains: participants, predictors, analysis, and outcome. Each domain contained 2-9 signaling questions, which could be answered as “yes or probably yes,” “no or probably no,” or “no information.” Domain-specific judgments were categorized as low, high, or unclear risk of bias. A domain was judged as having a low risk of bias if all signaling questions were answered “yes or probably yes”; a high risk of bias if at least one was answered “no or probably no”; or an unclear risk of bias if at least 1 was answered “no information” while all others were answered “yes or probably yes.” Two researchers (HS and ZX) conducted the assessment independently. They then cross-checked their results. Any disagreements were settled by consensus or arbitration by a third researcher (WW).

### Synthesis Methods

A bivariate random-effects model was used for the meta-analysis based on available 2×2 diagnostic tables (either reported directly or reconstructed from reported performance metrics and sample size). The following pooled estimates were derived with their corresponding 95% CIs, sensitivity, specificity, positive likelihood ratio (LR+), negative likelihood ratio (LR–), diagnostic odds ratio (DOR), and the area under the summary receiver operating characteristic curve (SROC AUC). Deeks’ funnel plot was used to evaluate small-study effects. Fagan’s nomogram was applied to evaluate clinical applicability. Subgroup analyses were conducted according to modeling variables (nonradiomic vs radiomic features) and model type (traditional machine learning vs deep learning). A *P* value of <.05 indicated statistical significance. Stata (version 15.1; StataCorp LLC) was used for all meta-analyses.

## Results

### Study Selection

**Figure 1:**
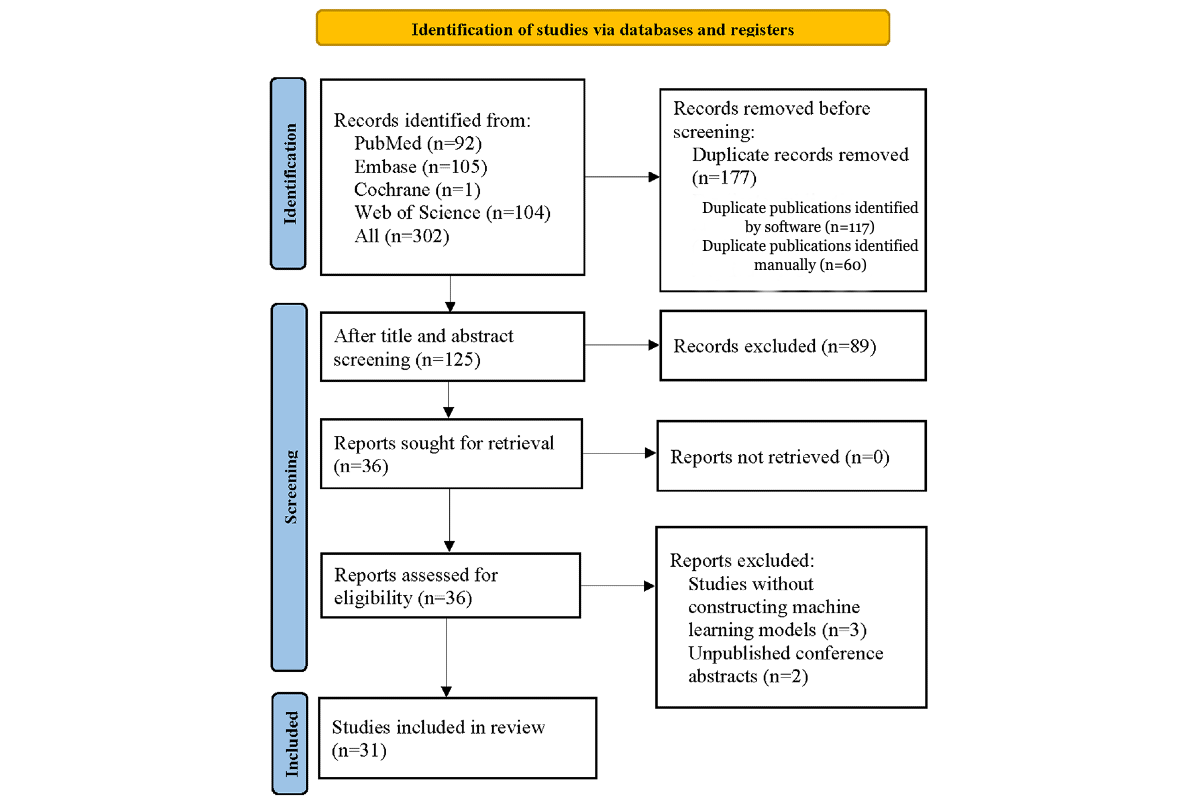
PRISMA (Preferred Reporting Items for a Systematic Review and Meta-Analysis) flow diagram of the selection process for studies applying machine learning to detect hepatocellular carcinoma vessels encapsulating tumor clusters.

The database search yielded 302 potentially relevant articles. Of these, 177 duplicates were excluded (117 identified by software and 60 manually). After screening the titles and abstracts, 89 articles unrelated to the topic were removed. The full texts of the remaining 36 articles were assessed for eligibility. Among them, 5 records were excluded; 3 because they did not develop machine learning models, and 2 because they were conference abstracts without full-text publication. Ultimately, 31 eligible studies were included [20-24,27-52]. The specific process is depicted in Figure 1.

### Study Characteristics

The 31 studies were published between 2021 and 2025. All were conducted in China and Japan. Of these, 8 studies used a case-control design, and 23 used a cohort design. Patient data were derived from multiple centers in 10 studies and from single centers in 21 studies. A total of 6755 participants with HCC were included, 2699 of whom were identified as VETC-positive. Regarding detection methods, 1 study used radiomic features based on computed tomography, 6 studies used MRI-based radiomics, 5 studies used deep learning, and 19 studies used traditional machine learning. The training sets collectively comprised 4411 participants with HCC, including 1714 with VETC. Internal validation was conducted in 14 studies, external validation in 3 studies, and both in 7 studies. The validation sets encompassed 2344 participants with HCC, 955 of whom were VETC-positive. The prediction models incorporated machine learning (n=5), logistic regression (n=24), least absolute shrinkage and selection operator regression (n=1), and random forest (n=1). Detailed characteristics are illustrated in Tables S2-S4 in Multimedia Appendix 2.

### Risk of Bias

**Figure 2:**
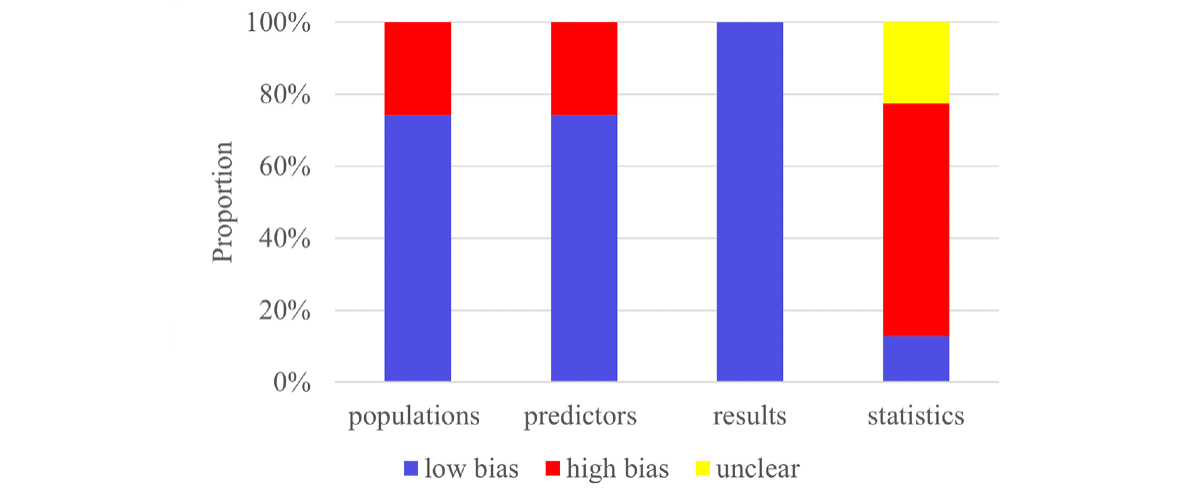
Risk of bias assessment for the included primary studies using the Prediction Model Risk of Bias Assessment Tool.

The Prediction Model Risk of Bias Assessment Tool was applied across 4 domains to assess the overall risk of bias. First, 8 of the 31 eligible studies in the participants domain used a case-control design, which introduced a high risk of bias due to potential differences in data sources and patient selection. Second, case-control studies were judged to carry a high risk of bias in the predictors domain because predictor assessment was influenced by knowledge of the outcome. Third, in the outcome domain, VETC status was consistently defined and confirmed via histopathological examination. Since the outcome definition, measurement, and classification were independent of predictor assessment and participant selection, this domain was assessed as having a low risk of bias. Fourth, in the analysis domain, 14 studies were judged to have a high risk of bias due to an insufficient sample size (including an events-per-variable ratio of <10 in model development, a validation set size of <100, or an absence of external validation). A total of 12 studies were rated as having an unclear risk of bias due to an inability to calculate the events-per-variable ratio. One study provided no explanation for missing values and was therefore judged to be at high risk of bias regarding missing data. Concerning model validation, 6 studies relied solely on random data splitting without cross-validation or mediator effect testing, resulting in a high risk of bias. Overall, 10 studies did not report the validation method used and were categorized as having an unclear risk of bias. Detailed assessment results are shown in Figure 2.

### Meta-Analysis

#### Training Set-Overall

A total of 27 models from the training sets provided 2×2 diagnostic tables, with a 39% VETC-positive proportion. The pooled estimates were as follows: sensitivity 0.77 (95% CI 0.72-0.82), specificity 0.83 (95% CI 0.78-0.87), LR+ 4.5 (95% CI 3.5-5.8), LR– 0.27 (95% CI 0.22-0.34), DOR 16 (95% CI 11-24), and SROC AUC 0.87 (95% CI 0.84-0.89; Figures S1 and S2 in Multimedia Appendix 2). No significant small-study effect was illustrated via Deeks’ funnel plot (*P*=.70; Figure S3 in Multimedia Appendix 2). Assuming a 40% a priori probability for the disease, the likelihood of an individual actually having VETC, given a VETC diagnosis by the model, was 75%. Conversely, the likelihood of an individual actually not having VETC, given a non-VETC diagnosis by the model, was 85% (Figure S4 in Multimedia Appendix 2).

#### Training Set-Nonradiomic Features

A total of 18 nonradiomic models from the training sets provided 2×2 diagnostic tables, with 38% representing VETC-positive cases. The pooled estimates were as follows: sensitivity 0.74 (95% CI 0.67-0.79), specificity 0.81 (95% CI 0.77-0.85), LR+ 3.9 (95% CI 3.2-4.7), LR– 0.33 (95% CI 0.27-0.40), DOR 12 (95% CI 9-16), and SROC AUC 0.85 (95% CI 0.81-0.88; Figures S5 and S6 in Multimedia Appendix 2). No significant small-study effect was detected via Deeks’ funnel plot (*P*=.46; Figure S7 in Multimedia Appendix 2). Assuming a 40% a priori probability for the disease, the likelihood of an individual actually having VETC, given a VETC diagnosis by the model, was 72%. Conversely, the likelihood of an individual actually not having VETC, given a non-VETC diagnosis by the model, was 82% (Figure S8 in Multimedia Appendix 2).

#### Training Set-Radiomic Features

A total of 9 radiomic models from the training set provided 2×2 diagnostic tables, with a VETC-positive rate of 40%. The pooled estimates were as follows: sensitivity 0.83 (95% CI 0.75-0.90), specificity 0.86 (95% CI 0.71-0.94), LR+ 6.0 (95% CI 2.6-13.5), LR– 0.19 (95% CI 0.11-0.32), DOR 31 (95% CI 9-106), and SROC AUC 0.91 (95% CI 0.88-0.93; Figures S9 and S10 in Multimedia Appendix 2). No significant small-study effect was observed via Deeks’ funnel plot (*P*=.40; Figure S11 in Multimedia Appendix 2). Assuming a 40% a priori probability for the disease, the likelihood of an individual actually having VETC, given a VETC diagnosis by the model, was 80%. Conversely, the likelihood of an individual actually not having VETC, given a non-VETC diagnosis by the model, was 89% (Figure S12 in Multimedia Appendix 2).

Of these, 6 traditional machine learning models provided 2×2 diagnostic tables, with a VETC-positive rate of 39%. The pooled estimates were as follows: sensitivity 0.88 (95% CI 0.70-0.96), specificity 0.85 (95% CI 0.67-0.94), LR+ 5.7 (95% CI 2.2-15.2), LR– 0.14 (95% CI 0.04-0.45), DOR 40 (95% CI 5-326), and SROC AUC 0.93 (95% CI 0.90-0.95; Figures S13 and S14 in Multimedia Appendix 2). No significant small-study effect was found via Deeks’ funnel plot (*P*=.78; Figure S15 in Multimedia Appendix 2). Assuming a 40% a priori probability for the disease, the likelihood of an individual actually having VETC, given a VETC diagnosis by the model, was 79%. Conversely, the likelihood of an individual actually not having VETC, given a non-VETC diagnosis by the model, was 91% (Figure S16 in Multimedia Appendix 2).

Only 3 deep learning studies reported 2×2 diagnostic tables. Yu et al [35] developed an MRI-based deep learning model with a sensitivity of 0.87, a specificity of 0.54, and an area under the receiver operating characteristic curve (ROC AUC) of 0.83 (95% CI 0.83-0.84). Xu et al [49] reported a contrast-enhanced ultrasound–based model with sensitivity of 0.75, specificity of 0.92, and ROC AUC of 0.92 (95% CI 0.88-0.96). Yang et al [48] developed an MRI-based model with a sensitivity of 0.71, a specificity of 0.97, and an ROC AUC of 0.90 (95% CI 0.85-0.95).

#### Validation Set-Overall

A total of 27 models in the validation set provided complete 2×2 diagnostic tables, with a VETC-positive proportion of 41%. The pooled estimates were as follows: sensitivity 0.77 (95% CI 0.72-0.81), specificity 0.74 (95% CI 0.69-0.78), LR+ 2.9 (95% CI 2.5-3.3), LR– 0.32 (95% CI 0.26-0.38), DOR 9 (95% CI 7-12), and SROC AUC 0.82 (95% CI 0.78-0.85; Figures S17 and S18 in Multimedia Appendix 2). Deeks’ funnel plot demonstrated no significant small-study effects (*P*=.09; Figure S19 in Multimedia Appendix 2). Assuming a 40% a priori probability for the disease, the likelihood of an individual actually having VETC, given a VETC diagnosis by the model, was 66%. Conversely, the likelihood of an individual actually not having VETC, given a non-VETC diagnosis by the model, was 83% (Figure S20 in Multimedia Appendix 2).

#### Validation Set-Nonradiomic Features

A total of 12 nonradiomic models in the validation set provided 2×2 diagnostic tables, with a VETC-positive proportion of 40%. The pooled estimates were as follows: sensitivity 0.72 (95% CI 0.66-0.78), specificity 0.74 (95% CI 0.68-0.80), LR+ 2.8 (95% CI 2.3-3.5), LR– 0.37 (95% CI 0.31-0.45), DOR 8 (95% CI 6-10), and SROC AUC 0.80 (95% CI 0.76-0.83; Figures S21 and S22 in Multimedia Appendix 2). No significant small-study effect was detected via Deeks’ funnel plot (*P*=.98; Figure S23 in Multimedia Appendix 2). Assuming a 40% a priori probability for the disease, the likelihood of an individual actually having VETC, given a VETC diagnosis by the model, was 65%. Conversely, the likelihood of an individual actually not having VETC, given a non-VETC diagnosis by the model, was 80% (Figure S24 in Multimedia Appendix 2).

#### Validation Set-Radiomic Features

A total of 15 radiomic models in the validation set provided 2×2 diagnostic tables, with a VETC-positive rate of 41%. The pooled estimates were as follows: sensitivity 0.81 (95% CI 0.73-0.87), specificity 0.73 (95% CI 0.67-0.79), LR+ 3.0 (95% CI 2.5-3.7), LR– 0.26 (95% CI 0.19-0.36), DOR 12 (95% CI 8-17), and SROC AUC 0.84 (95% CI 0.80-0.87; Figures S25 and S26 in Multimedia Appendix 2). No significant small-study effect was observed via Deeks’ funnel plot (*P*=.11; Figure S27 in Multimedia Appendix 2). Assuming a 40% a priori probability for the disease, the likelihood of an individual actually having VETC, given a VETC diagnosis by the model, was 67%. Conversely, the likelihood of an individual actually not having VETC, given a non-VETC diagnosis by the model, was 85% (Figure S28 in Multimedia Appendix 2).

Of these, 9 traditional machine learning models provided 2×2 diagnostic tables, with a VETC-positive rate of 41%. The pooled estimates were as follows: sensitivity 0.84 (95% CI 0.71-0.92), specificity 0.75 (95% CI 0.67-0.81), LR+ 3.3 (95% CI 2.6-4.3), LR– 0.21 (95% CI 0.11-0.39), DOR 16 (95% CI 8-32), and SROC AUC 0.83 (95% CI 0.80-0.86; Figure S29 and S30 in Multimedia Appendix 2). No significant small-study effect was shown via Deeks’ funnel plot (*P*=.37; Figures S31 in Multimedia Appendix 2). Assuming a 40% a priori probability for the disease, the likelihood of an individual actually having VETC, given a VETC diagnosis by the model, was 69%. Conversely, the likelihood of an individual actually not having VETC, given a non-VETC diagnosis by the model, was 88% (Figure S32 in Multimedia Appendix 2).

Additionally, 6 deep learning models reported 2×2 diagnostic tables, with a VETC-positive proportion of 41%. The pooled estimates were as follows: sensitivity 0.77 (95% CI 0.69-0.84), specificity 0.70 (95% CI 0.59-0.79), LR+ 2.6 (95% CI 1.9-3.5), LR– 0.32 (95% CI 0.24-0.43), DOR 8 (95% CI 5-13), and SROC AUC 0.81 (95% CI 0.77-0.85; Figures S33 and S34 in Multimedia Appendix 2). Deeks’ funnel plot suggested significant small-study effects (*P*=.04; Figure S35 in Multimedia Appendix 2). Assuming a 40% a priori probability for the disease, the likelihood of an individual actually having VETC, given a VETC diagnosis by the model, was 63%. Conversely, the likelihood of an individual actually not having VETC, given a non-VETC diagnosis by the model, was 82% (Figure S36 in Multimedia Appendix 2).

## Discussion

### Summary of Main Findings

This meta-analysis demonstrates that developing prediction models based on machine learning to detect HCC VETC status appears to be a feasible approach. Currently, these models are primarily constructed using nonradiomic and radiomic features. For nonradiomic machine learning models in the validation set, the pooled estimates were 0.72 (95% CI 0.66-0.78) for sensitivity and 0.74 (95% CI 0.68-0.80) for specificity. For radiomic machine learning models, the estimates were a sensitivity of 0.81 (95% CI 0.73-0.87) and a specificity of 0.73 (95% CI 0.67-0.79). For traditional machine learning models, the estimates were a sensitivity of 0.84 (95% CI 0.71-0.92) and a specificity of 0.75 (95% CI 0.67-0.81). For deep learning models, the estimates were a sensitivity of 0.77 (95% CI 0.69-0.84) and a specificity of 0.70 (95% CI 0.59-0.79).

### Comparison With Previous Reviews

Previous research by Hyungjin Rhee et al [53] reviewed the angiodynamic changes in multistep HCC carcinogenesis. They introduced the typical pathological, clinical, and imaging features of HCC VETC and provided detailed guidance for VETC diagnosis. However, their study focused primarily on describing pathological mechanisms and typical features, lacking a quantitative assessment of different diagnostic methods. Ken Liu et al [9] investigated various methods for diagnosing VETC, including histopathology, imaging, and laboratory tests. They suggested that VETC could be predicted radiologically. While their research provided a comprehensive analysis of various diagnostic approaches, they did not quantitatively compare the sensitivity and specificity of different diagnostic methods. This omission limited a thorough evaluation of VETC diagnostic accuracy. Miaomiao Wang et al [54] explored the potential of machine learning in HCC VETC detection through a literature review and provided guidance for the auxiliary VETC diagnosis. While their review demonstrated the potential applications of machine learning in VETC detection, it lacked a direct comparison of different types of machine learning models, making it difficult to assess these models’ actual application value in clinical practice. This study summarized nonradiomic (clinical features, image features, etc) and radiomic prediction models, and the diagnosis of current HCC VETC status appears to be an ideal noninvasive detection scheme that provides specific guidance for clinicians.

This study found that the model variables used to detect HCC VETC include both nonradiomic features (clinical features, image features, etc) and radiomic features. The clinical features primarily consist of alpha-fetoprotein, carbohydrate antigen 19-9, aspartate aminotransferase, and indirect bilirubin. Image features mainly comprise intratumoral necrosis, low signal intensity around the tumor in the hepatobiliary phase, the tumor-to-liver signal intensity ratio on the hepatobiliary phase, and the tumor-to-liver apparent diffusion coefficient ratio. Various studies used different modeling variables. Most studies did not quantitatively present the association of modeling variables with VETC. Thus, a further summary of such correlations was not performed. Recently, radiomics has advanced the development and application of prediction models by converting images into repeatable quantitative data. Prediction models based on radiomic features have demonstrated significant clinical value in diagnosing and treating HCC. Studies have shown that radiomic features are effective in predicting HCC microvascular invasion [5], early recurrence [55], and Ki-67 and cytokeratin 19 expression [7].

In this meta-analysis, only a limited number of studies explored the diagnostic performance of radiomics for HCC VETC. While the studies demonstrated promising results, radiomics still faces significant challenges in practical application. For example, the quality of the image appears to change under different image parameters. Most studies in this meta-analysis did not discuss how such changes in image features affect radiomics results. Additionally, image segmentation is primarily divided into manual and deep learning automatic segmentation. The studies included in this meta-analysis primarily used manual segmentation. However, manual segmentation may be affected by the segmenter’s prior knowledge. Although some researchers have attempted to summarize its repeatability through independent segmentation by multiple people, it is difficult to avoid the influence of the segmenter’s experience on the region-of-interest area division. Therefore, future studies should consider developing and promoting a standardized radiomics analysis process manual to improve research repeatability. Many studies have demonstrated that models combining radiomics, clinical features, and imaging features perform better in disease diagnosis and prognosis prediction [56]. In this study, relatively few studies attempted to construct prediction models using a combination of clinical features and radiomics. Therefore, an effective quantitative analysis of the advantages of a combined model was difficult to perform. Future studies should explore and verify the value of radiomic models constructed from clinical features and imaging features in improving the diagnostic accuracy of HCC VETC.

The prediction models used in this study primarily encompassed logistic regression, random forest, deep learning, and least absolute shrinkage and selection operator regression. Due to the interpretability of its parameters, logistic regression allows for the development of simple and intuitive nomograms in clinical practice and appears to be favored by many researchers [57-59]. However, the interpretability of other machine learning models, such as random forest, support vector machines, and XGBoost, depends on analyses like Shapley additive explanations. Using them in clinical practice requires developing plugins, which increases the complexity of the application process [60-62]. Thus, from the perspectives of clinical simplicity and interpretability, logistic regression has relatively ideal advantages. Nonetheless, in many cases, logistic regression’s predictive accuracy often appears no better than that of traditional machine learning models, such as random forest [46,63]. In radiomics, the core advantage of deep learning lies in its ability to efficiently process image data for disease diagnosis and prognosis prediction [64,65]. Relatively few studies in the radiomic feature literature included in this meta-analysis addressed deep learning models. Initial evidence suggested that deep learning models did not perform significantly better than traditional machine learning models. The primary reasons for this include the following. First, the study only incorporated 6 deep learning research projects, which is a relatively small sample size. Deep learning models typically require large-scale datasets to leverage their full advantages. Second, most studies lacked external validation, leaving the generalizability of the models inadequately tested. Third, variations in image acquisition parameters and quality across different research centers suggest that the design of deep learning model architectures and hyperparameter optimization may not yet be optimal. Therefore, future research developing intelligent tools to detect HCC VETC should attempt to integrate multicenter, large-sample medical image data to construct deep learning models for training and validation.

### Advantages and Limitations

This meta-analysis is the first comprehensive summary of the performance of machine learning models in diagnosing HCC VETC. It provides evidence-based support for the subsequent development or updating of artificial intelligence systems. However, this study also has the following limitations. First, all 31 eligible studies originated from East Asia, and most relied primarily on internal validation. The lack of multicenter, multiethnic validation limited the assessment of the models’ generalizability. Second, the best prediction model from each article was extracted, which covered a narrow range of machine learning types. The differences between different machine learning methods were not described. Third, the modeling variables were diverse. They were only presented without a quantitative description of their association with HCC VETC. Future research should adopt more transparent and interpretable modeling approaches to identify efficient predictors. Fourth, although deep learning can efficiently process image data, it does not have a significant advantage over traditional machine learning-based radiomics. However, the literature is limited, and the interpretation of the results may be subject to certain limitations. Fifth, HCC VETC is a novel mode of microvascular metastasis that has been proposed in recent years, and the associated research is in its initial stage. The positive definition has not yet been standardized.

### Conclusions

This meta-analysis is the first to provide a systematic and quantitative assessment of machine learning for diagnosing HCC VETC, thereby addressing an evidence gap in this field. Unlike previous reviews, this study provides a quantitative evaluation of diagnostic performance. The findings demonstrate the feasibility and clinical potential of using machine learning to determine VETC status in patients with HCC. Notably, radiomics-based models exhibited significantly better performance than nonradiomic models. While deep learning efficiently processes image data in radiomics, its performance is not significantly better than traditional machine learning-based radiomics. Despite their promising prospects, machine learning models have not yet reached the maturity required for clinical translation, owing to methodological heterogeneity, limited validation, and a high risk of bias. Future research should focus on conducting multicenter, large-sample, standardized, prospective studies to develop intelligent detection tools with higher performance. Validating the models across multiple regions and ethnic populations is essential to ensure their generalizability. This will ultimately enable the effective translation of research into clinical applications.

## Appendix

Multimedia Appendix 1 PRISMA-S checklist.

Multimedia Appendix 2 Supplementary documents.

## Abbreviations

DOR: diagnostic odds ratio
HCC: hepatocellular carcinoma
LR–: negative likelihood ratio
LR+: positive likelihood ratio
MRI: magnetic resonance imaging
PRISMA: Preferred Reporting Items for a Systematic Review and Meta-Analysis
ROC AUC: area under the receiver operating characteristic curve
SROC AUC: area under the summary receiver operating characteristic curve
VETC: vessels encapsulating tumor clusters

## Data Availability

All data used in this study are included in the main text and supplementary materials. For further information or clarification, please contact the corresponding researchers.
