# Supplementary material for: Effectiveness of Machine Learning in Detecting Vessels Encapsulating Tumor Clusters in Hepatocellular Carcinoma: Systematic Review and Meta-Analysis
Source: J Med Internet Res. 2026 Jan 14;28:e82839. doi: 10.2196/82839 (PMC12853091; doi:10.2196/82839)
Supplement: Multimedia Appendix 2 [file jmir_v28i1e82839_app2.docx]

Table S1 Literature retrieval strategy for machine learning in the detection of VETC in hepatocellular carcinoma

**1.Pubmed**

| Search number | Query | Results |
| --- | --- | --- |
| #1 | "Carcinoma, Hepatocellular"[Mesh] | 115165 |
| #2 | (((((((((((((((((hepatic carcinoma[Title/Abstract]) ) OR (hepatic cell carcinoma[Title/Abstract])) OR (hepatocarcinoma[Title/Abstract])) OR (Hepatocellular Carcinoma[Title/Abstract])) OR (Hepatocellular Carcinomas[Title/Abstract])) OR (hepatocellular carcinomata[Title/Abstract])) OR (hepatocyte carcinoma[Title/Abstract])) OR (hepatocytic carcinoma[Title/Abstract])) OR (Hepatoma[Title/Abstract])) OR (Hepatomas[Title/Abstract])) OR (hepatomata[Title/Abstract])) OR (hepatomatous[Title/Abstract])) OR (Liver Cancer[Title/Abstract])) OR (Liver Cancers[Title/Abstract])) OR (liver carcinoma[Title/Abstract])) OR (Liver Cell Carcinoma[Title/Abstract])) OR (Liver Cell Carcinomas[Title/Abstract]) | 191082 |
| #3 | #1 OR #2 | 206700 |
| #4 | (((Vessels Encapsulating Tumor Clusters[Title/Abstract]) OR (VETC[Title/Abstract])) OR (vessels that encapsulate tumor clusters[Title/Abstract])) OR (vascular wrapping around tumor clusters[Title/Abstract]) | 99 |
| #5 | #3 AND #4 | 92 |

**2.Cochrane**

| Search number | Query | Results |
| --- | --- | --- |
| #1 | MeSH descriptor: [Carcinoma, Hepatocellular] explode all trees | 2810 |
| #2 | (hepatic carcinoma): ti, ab, kw OR (hepatic cell carcinoma): ti, ab, kw OR (hepatocarcinoma): ti, ab, kw OR (Hepatocellular Carcinoma): ti, ab,kw OR (Hepatocellular Carcinomas): ti, ab, kw | 7737 |
| #3 | (hepatocellular carcinomata): ti, ab, kw OR (hepatocyte carcinoma): ti, ab, kw OR (hepatocytic carcinoma): ti, ab, kw OR (Hepatoma): ti, ab, kw OR (Hepatomas): ti, ab, kw | 371 |
| #4 | (hepatomata): ti, ab ,kw OR (hepatomatous): ti, ab, kw OR (Liver Cancer): ti, ab, kw OR (Liver Cancers): ti, ab, kw OR (liver carcinoma): ti, ab, kw | 17754 |
| #5 | (Liver Cell Carcinoma): ti, ab, kw OR (Liver Cell Carcinomas): ti, ab, kw | 4541 |
| #6 | #1 OR #2 OR #3 OR #4 OR #5 | 19621 |
| #7 | (Vessels Encapsulating Tumor Clusters): ti, ab, kw OR (VETC): ti, ab, kw OR (vessels that encapsulate tumor clusters): ti, ab, kw OR (vascular wrapping around tumor clusters): ti, ab, kw | 5 |
| #8 | #6 AND #7 | 1 |

**3.Embase**

| Search number | Query | Results |
| --- | --- | --- |
| #1 | 'liver cell carcinoma'/exp | 241534 |
| #2 | 'hepatic carcinoma': ab, ti OR hepatocarcinoma: ab, ti OR 'hepatocellular carcinoma': ab, ti OR 'hepatocellular carcinomas':ab,ti OR 'hepatocellular carcinomata':ab,ti OR 'hepatocyte carcinoma':ab,ti OR 'hepatocytic carcinoma':ab,ti OR hepatoma:ab,ti OR hepatomas:ab,ti OR hepatomata:ab,ti OR hepatomatous:ab,ti OR 'liver cancer':ab,ti OR 'liver cancers':ab,ti OR 'liver carcinoma':ab,ti OR 'liver cell carcinoma':ab,ti OR 'liver cell carcinomas':ab,ti | 261818 |
| #3 | #1 OR #2 | 312958 |
| #4 | 'vessels encapsulating tumor clusters':ab,ti OR vetc:ab,ti OR 'vessels that encapsulate tumor clusters':ab,ti OR 'vascular wrapping around tumor clusters':ab,ti | 119 |
| #5 | #3 AND #4 | 105 |

**4.Web of science**

| Search number | Query | Results |
| --- | --- | --- |
| #1 | VETC (Topic) OR Vessels Encapsulating Tumor Clusters (Topic) OR vessels that encapsulate tumor clusters (Topic) OR vascular wrapping around tumor clusters (Topic) | 120 |
| #2 | Carcinoma, Hepatocellular (Topic) OR hepatic carcinoma (Topic) OR hepatic cell carcinoma (Topic) OR hepatocarcinoma (Topic) OR Hepatocellular Carcinoma (Topic) OR Hepatocellular Carcinomas (Topic) OR hepatocellular carcinomata (Topic) OR hepatocyte carcinoma (Topic) OR hepatocytic carcinoma (Topic) OR Hepatoma (Topic) OR Hepatomas (Topic) OR hepatomata (Topic) OR hepatomatous (Topic) OR Liver Cancer (Topic) OR Liver Cancers (Topic) OR liver carcinoma (Topic) OR Liver Cell Carcinoma (Topic) OR Liver Cell Carcinomas (Topic) | 338700 |
| #3 | #1 AND #2 | 104 |

Table S2 Basic characteristics of 31 included studies on machine learning for detecting VETC in hepatocellular carcinoma published from 2021 to 2025

| No. | First author | Year of publication | Country of author | Study type | Patient source | Number of VETC cases | Total number of cases | Detection method | The number of VETC cases in the training set | Total number of cases in the training set | The generation method of the validation set | The number of VETC cases in the validation set | Total number of cases in the validation set | Model type |
| --- | --- | --- | --- | --- | --- | --- | --- | --- | --- | --- | --- | --- | --- | --- |
| 1 | Qi Qu | 2024 | China | Cohort study | Single center | 105 | 240 | Machine learning | 74 | 168 | random sampling 10-fold cross-validation | 31 | 72 | LR |
| 2 | Huilin Chen | 2024 | China | Case-control study | Multicenter | 138 | 252 | Machine learning | 82 | 142 | Bootstrap external validation | set1:33 set1:23 | set1:64 set1:46 | LR |
| 3 | Min Li | 2025 | China | Cohort study | Single center | 29 | 116 | Machine learning | 29 | 116 | None | None | None | LR |
| 4 | Tongjia Chu | 2022 | China | Case-control study | Single center | 44 | 133 | Deep learning | 31 | 93 | random sampling | 13 | 40 | DL |
| 5 | Feng Che | 2025 | China | Cohort study | Multicenter | 170 | 505 | Radiomics | 82 | 253 | random sampling 5-fold cross-validation external validation | set1:34 set2:54 | set1:108 set2:144 | LR |
| 6 | HuiLin Chen | 2024 | China | Cohort study | Multicenter | 155 | 309 | Machine learning | 86 | 177 | Bootstrap external validation | set1:41 set2:28 | set1:78 set2:54 | LR |
| 7 | Yanfen Fan | 2021 | China | Case-control study | Single center | 52 | 133 | Radiomics | 52 | 133 | 5-fold cross-validation | None | None | LR |
| 8 | Chao Zhang | 2024 | China | Case-control study | Multicenter | 94 | 190 | Radiomics | 53 | 106 | random sampling 10-fold cross-validation external validation | set1:23 set2:18 | set1:47 set2:37 | LR |
| 9 | Yanfen Fan | 2021 | China | Case-control study | Single center | 47 | 109 | Machine learning | 47 | 109 | None | None | None | LR |
| 10 | Yixing Yu | 2025 | China | Cohort study | Multicenter | 299 | 578 | Deep learning | 164 | 317 | random sampling 10-fold cross-validation external validation | set1:71 set2:64 | set1:137 set2:124 | DL |
| 11 | Qianjiang Ding | 2025 | China | Cohort study | Single center | 11 | 53 | Machine learning | 11 | 53 | None | None | None | LR |
| 12 | Fang Ming Chen | 2023 | China | Cohort study | Multicenter | 156 | 320 | Machine learning | 83 | 173 | bootstrapp external validation | 73 | 147 | LR |
| 13 | Feiqian Wang | 2024 | Japan | Cohort study | Single center | 21 | 101 | Machine learning | 21 | 101 | Bootstrap | None | None | LR |
| 14 | Jiyun Zhang | 2024 | China | Cohort study | Single center | 101 | 234 | Radiomics | 71 | 163 | random sampling 5-fold cross-validation | 30 | 71 | LR |
| 15 | Zhichao Feng | 2021 | China | Cohort study | Multicenter | 74 | 271 | Machine learning | 52 | 170 | external validation | 22 | 101 | LR |
| 16 | Kosuke Matsuda | 2025 | Japan | Cohort study | Single center | 45 | 204 | Radiomics | 33 | 153 | random sampling | 12 | 51 | LASSO |
| 17 | Jiawen Yang | 2025 | China | Cohort study | Multicenter | 144 | 320 | Radiomics | 61 | 153 | random sampling external validation | set1:27 set2:56 | set1:66 set2:101 | LR |
| 18 | Junhan Pan | 2025 | China | Case-control study | Single center | 99 | 324 | Machine learning | 69 | 227 | random sampling 5-fold cross-validation | 30 | 97 | LR |
| 19 | Dong Xue | 2024 | China | Cohort study | Single center | 68 | 221 | Deep learning | 46 | 154 | The operation date is not random. | 22 | 67 | DL |
| 20 | Yixing Yu | 2022 | China | Cohort study | Single center | 97 | 182 | Radiomics | 72 | 128 | random sampling 5-fold cross-validation | 25 | 54 | RF |
| 21 | Renguo Guan | 2022 | China | Cohort study | Single center | 87 | 365 | Machine learning | 58 | 243 | random sampling | 29 | 122 | LR |
| 22 | Wenxin Xu | 2025 | China | Cohort study | Single center | 96 | 242 | Deep learning | 80 | 195 | random sampling | 16 | 47 | DL |
| 23 | XiangPan Meng | 2025 | China | Cohort study | Multicenter | 94 | 191 | Machine learning | None | None | external validation | 94 | 191 | LR |
| 24 | Zongwen Li | 2024 | China | Case-control study | Single center | 15 | 36 | Machine learning | 15 | 36 | Bootstrap | None | None | LR |
| 25 | Litao Ruan | 2024 | China | Case-control study | Single center | 81 | 215 | Machine learning | 51 | 129 | random sampling cross validation | 30 | 86 | LR |
| 26 | Yinzhong Wang | 2024 | China | Cohort study | Single center | 37 | 84 | Machine learning | 37 | 84 | None | None | None | LR |
| 27 | Chenhui Li | 2023 | China | Cohort study | Single center | 40 | 86 | Machine learning | 40 | 86 | None | None | None | LR |
| 28 | Yongquan Yu | 2025 | China | Cohort study | Single center | 21 | 50 | Machine learning | 21 | 50 | None | None | None | LR |
| 29 | Jiawen Yang | 2024 | China | Cohort study | Multicenter | 93 | 320 | Deep learning | 68 | 219 | external validation | 25 | 101 | DL |
| 30 | Wenxin Xu | 2024 | China | Cohort study | Single center | 109 | 273 | Machine learning | 78 | 182 | random sampling | 31 | 91 | LR |
| 31 | Miaomiao Wang | 2024 | China | Cohort study | Single center | 47 | 98 | Machine learning | 47 | 98 | None | None | None | LR |

Table S3 Outcome indicators for machine learning in the detection of VETC in hepatocellular carcinoma in the training set

| Study | Data_set | Events | Sample size | Model | Variable | Sensitivity | Specificity | tp | fp | fn | tn |
| --- | --- | --- | --- | --- | --- | --- | --- | --- | --- | --- | --- |
| Qi Qu(2024) | Training set | 74 | 168 | LR | Clinical features | 0.8380 | 0.8380 | 62 | 15 | 12 | 79 |
| Huilin Chen(2024) | Training set | 82 | 142 | LR | Clinical features | 0.7680 | 0.8330 | 63 | 10 | 19 | 50 |
| Min Li(2025) | Training set | 29 | 116 | LR | Clinical features | 0.8620 | 0.7360 | 25 | 23 | 4 | 64 |
| Feng Che(2025) | Training set | 82 | 253 | LR | Radiomics | 0.8300 | 0.7500 | 68 | 43 | 14 | 128 |
| HuiLin Chen(2024) | Training set | 86 | 177 | LR | Clinical features | 0.6740 | 0.8130 | 58 | 17 | 28 | 74 |
| Yanfen Fan(2021) | Training set | 52 | 133 | LR | Radiomics | 0.8080 | 0.7410 | 42 | 21 | 10 | 60 |
| Chao Zhang(2024) | Training set | 53 | 106 | LR | Radiomics | 0.8300 | 0.7540 | 44 | 13 | 9 | 40 |
| Yanfen Fan(2021) | Training set | 47 | 109 | LR | Clinical features | 0.8390 | 0.8090 | 39 | 12 | 8 | 50 |
| Yixing Yu(2025) | Training set | 164 | 317 | DL | MRI | 0.8670 | 0.5390 | 142 | 71 | 22 | 82 |
| Qianjiang Ding(2025) | Training set | 11 | 53 | LR | Clinical features | 0.8180 | 0.8810 | 9 | 5 | 2 | 37 |
| Fangming Chen(2023) | Training set | 83 | 173 | LR | Clinical features | 0.7470 | 0.8890 | 62 | 10 | 21 | 80 |
| Feiqian Wang(2024) | Training set | 21 | 101 | LR | Clinical features | 0.3750 | 0.9430 | 8 | 5 | 13 | 75 |
| Jiyun Zhang(2024) | Training set | 71 | 163 | LR | Radiomics | 0.7857 | 0.8191 | 56 | 17 | 15 | 75 |
| Zhichao Feng(2021) | Training set | 52 | 170 | LR | Clinical features | 0.6000 | 0.7900 | 31 | 25 | 21 | 93 |
| Kosuke Matsuda(2025) | Training set | 33 | 153 | LASSO | Radiomics | 0.7415 | 0.7787 | 24 | 27 | 9 | 93 |
| Junhan Pan(2025) | Training set | 69 | 227 | LR | Clinical features | 0.8700 | 0.8230 | 60 | 28 | 9 | 130 |
| Yixing Yu(2022) | Training set | 72 | 128 | RF | Radiomics | 1.0000 | 1.0000 | 72 | 0 | 0 | 56 |
| Renguo Guan(2022) | Training set | 58 | 243 | LR | Clinical features | 0.5861 | 0.7930 | 34 | 38 | 24 | 147 |
| Wenxin Xu(2025) | Training set | 80 | 195 | DL | CEUS | 0.7500 | 0.9200 | 60 | 9 | 20 | 106 |
| Zongwen Li(2024) | Training set | 15 | 36 | LR | Clinical features |  |  | 11 | 0 | 4 | 21 |
| Litao Ruan(2024) | Training set | 51 | 129 | LR | Clinical features | 0.8820 | 0.6280 | 45 | 29 | 6 | 49 |
| Yinzhong Wang(2024) | Training set | 37 | 84 | LR | Clinical features | 0.7838 | 0.6809 | 29 | 15 | 8 | 32 |
| Chenhui Li(2023) | Training set | 40 | 86 | LR | Clinical features | 0.7000 | 0.6960 | 28 | 14 | 12 | 32 |
| Yongquan Yu(2025) | Training set | 21 | 50 | LR | Clinical features | 0.6667 | 0.8966 | 14 | 3 | 7 | 26 |
| Jiawen Yang(2024) | Training set | 68 | 219 | DL | MRI | 0.7100 | 0.9700 | 48 | 5 | 20 | 146 |
| Wenxin Xu(2024) | Training set | 78 | 182 | LR | Clinical features | 0.5900 | 0.8200 | 46 | 19 | 32 | 85 |
| Miaomiao Wang(2024) | Training set | 47 | 98 | LR | Clinical features | 0.7174 | 0.6800 | 34 | 16 | 13 | 35 |

Table S4 Outcome indicators for machine learning in the detection of VETC in hepatocellular carcinoma in the validation set

| No | Study | Data_set | Events | Sample size | Model | Variable | Sensitivity | Specificity | tp | fp | fn | tn |
| --- | --- | --- | --- | --- | --- | --- | --- | --- | --- | --- | --- | --- |
| 1 | Qi Qu(2024) | Validation set | 31 | 72 | LR | Clinical features | 0.7270 | 0.7270 | 23 | 11 | 8 | 30 |
| 2 | Huilin Chen(2024) | Validation set | 33 | 64 | LR | Clinical features | 0.7270 | 0.8390 | 24 | 5 | 9 | 26 |
| 2 | Huilin Chen(2024) | Validation set | 23 | 46 | LR | Clinical features | 0.8700 | 0.6520 | 20 | 8 | 3 | 15 |
| 4 | Tongjia Chu(2022) | Validation set | 13 | 40 | DL | MRI | 0.8376 | 0.7060 | 11 | 8 | 2 | 19 |
| 5 | Feng Che(2025) | Validation set | 34 | 108 | LR | Radiomics | 0.7700 | 0.7200 | 26 | 21 | 8 | 53 |
| 5 | Feng Che(2025) | Validation set | 54 | 144 | LR | Radiomics | 0.8000 | 0.7000 | 43 | 27 | 11 | 63 |
| 6 | Huilin Chen(2024) | Validation set | 41 | 78 | LR | Clinical features | 0.6830 | 0.8110 | 28 | 7 | 13 | 30 |
| 6 | Huilin Chen(2024) | Validation set | 28 | 54 | LR | Clinical features | 0.7500 | 0.7690 | 21 | 6 | 7 | 20 |
| 8 | Chao Zhang(2024) | Validation set | 23 | 47 | LR | Radiomics | 0.8260 | 0.7500 | 19 | 6 | 4 | 18 |
| 8 | Chao Zhang(2024) | Validation set | 18 | 37 | LR | Radiomics | 0.6110 | 0.8420 | 11 | 3 | 7 | 16 |
| 10 | Yixing Yu(2025) | Validation set | 71 | 137 | DL | MRI | 0.8580 | 0.4790 | 61 | 34 | 10 | 32 |
| 10 | Yixing Yu(2025) | Validation set | 64 | 124 | DL | MRI | 0.6540 | 0.7910 | 42 | 13 | 22 | 47 |
| 12 | Fangming Chen(2023) | Validation set | 73 | 147 | LR | Clinical features | 0.7360 | 0.8130 | 54 | 14 | 19 | 60 |
| 14 | Jiyun Zhang(2024) | Validation set | 30 | 71 | LR | Radiomics | 1.0000 | 0.5128 | 30 | 20 | 0 | 21 |
| 15 | Zhichao Feng(2021) | Validation set | 22 | 101 | LR | Clinical features | 0.7300 | 0.5400 | 16 | 36 | 6 | 43 |
| 16 | Kosuke Matsuda(2025) | Validation set | 12 | 51 | LASSO | Radiomics | 0.8388 | 0.7708 | 10 | 9 | 2 | 30 |
| 17 | Jiawen Yang(2025) | Validation set | 27 | 66 | LR | Radiomics | 0.8900 | 0.7400 | 24 | 10 | 3 | 29 |
| 17 | Jiawen Yang(2025) | Validation set | 56 | 101 | LR | Radiomics | 0.5800 | 0.8800 | 32 | 5 | 24 | 40 |
| 18 | Junhan Pan(2025) | Validation set | 30 | 97 | LR | Clinical features | 0.8670 | 0.7460 | 26 | 17 | 4 | 50 |
| 19 | Dong Xue(2024) | Validation set | 22 | 67 | DL | MRI | 0.7730 | 0.8220 | 17 | 8 | 5 | 37 |
| 20 | Yixing Yu(2022) | Validation set | 25 | 54 | RF | Radiomics | 1.0000 | 0.8621 | 25 | 4 | 0 | 25 |
| 21 | Renguo Guan(2022) | Validation set | 29 | 122 | LR | Clinical features | 0.6207 | 0.6756 | 18 | 30 | 11 | 63 |
| 22 | Wenxin Xu(2025) | Validation set | 16 | 47 | DL | CEUS | 0.8100 | 0.8100 | 13 | 6 | 3 | 25 |
| 23 | XiangPan Meng(2025) | Validation set | 94 | 191 | LR | Clinical features | 0.7020 | 0.7630 | 66 | 23 | 28 | 74 |
| 25 | Litao Ruan(2024) | Validation set | 30 | 86 | LR | Clinical features | 0.7670 | 0.6430 | 23 | 20 | 7 | 36 |
| 29 | Jiawen Yang(2024) | Validation set | 25 | 101 | DL | MRI | 0.8000 | 0.6100 | 20 | 30 | 5 | 46 |
| 30 | Wenxin Xu(2024) | Validation set | 31 | 91 | LR | Clinical features | 0.3900 | 0.9000 | 12 | 6 | 19 | 54 |


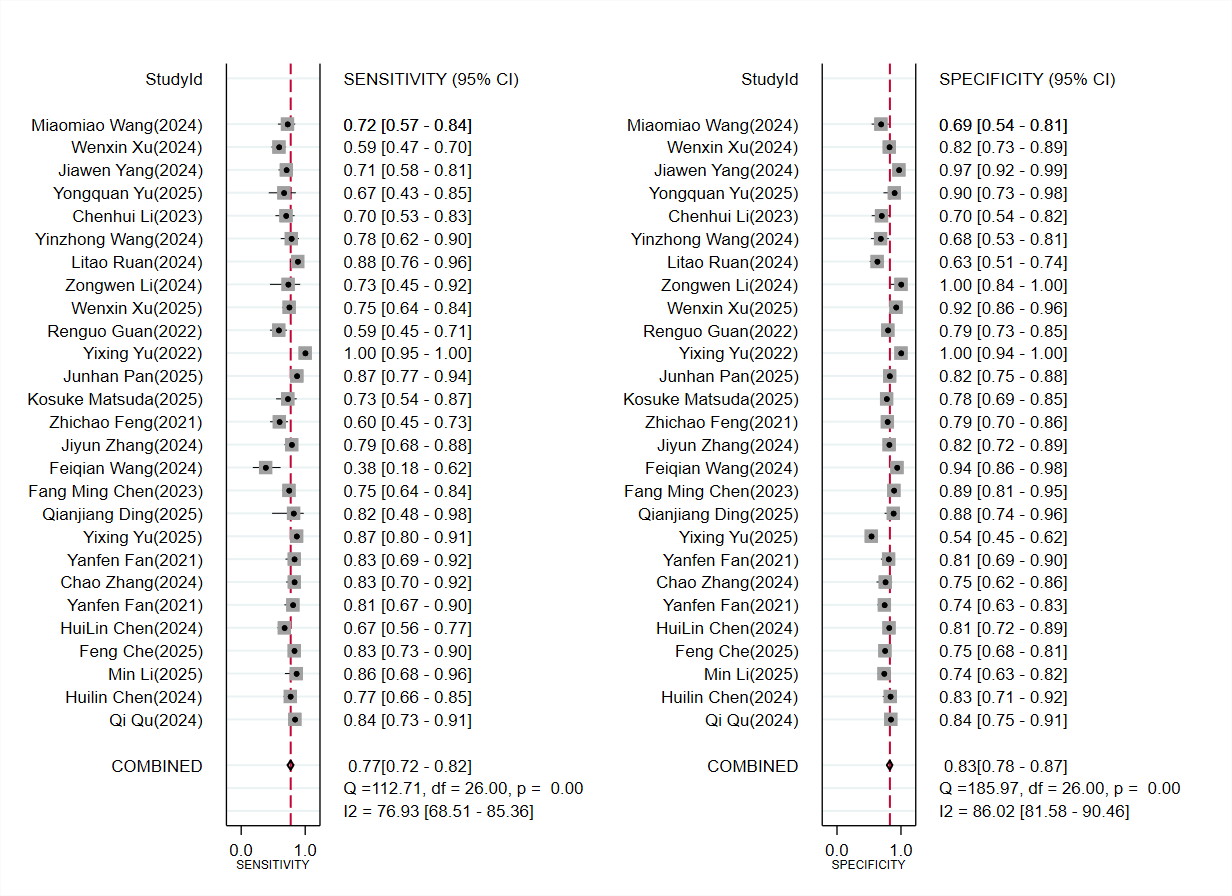


Figure S1 Forest plot from meta-analysis assessing the sensitivity and specificity of all machine learning models for VETC diagnosis in the training set


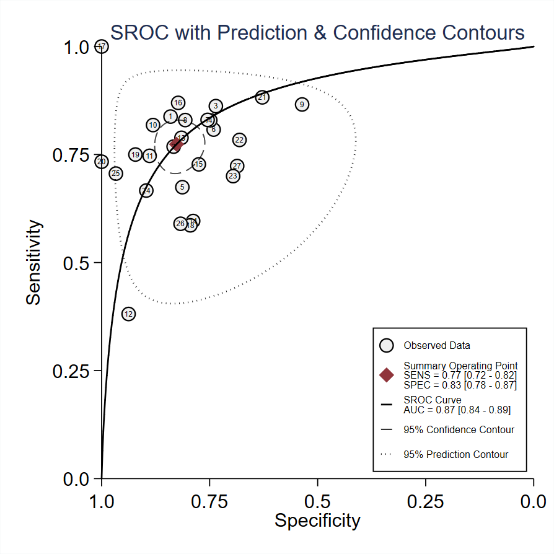


Figure S2 SROC curve from meta-analysis evaluating all machine learning models in VETC diagnosis in the training set


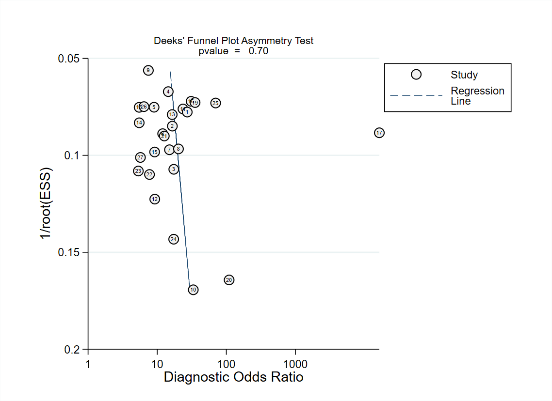


Figure S3 Deeks' funnel plot from meta-analysis examining all machine learning models for VETC diagnosis in the training set


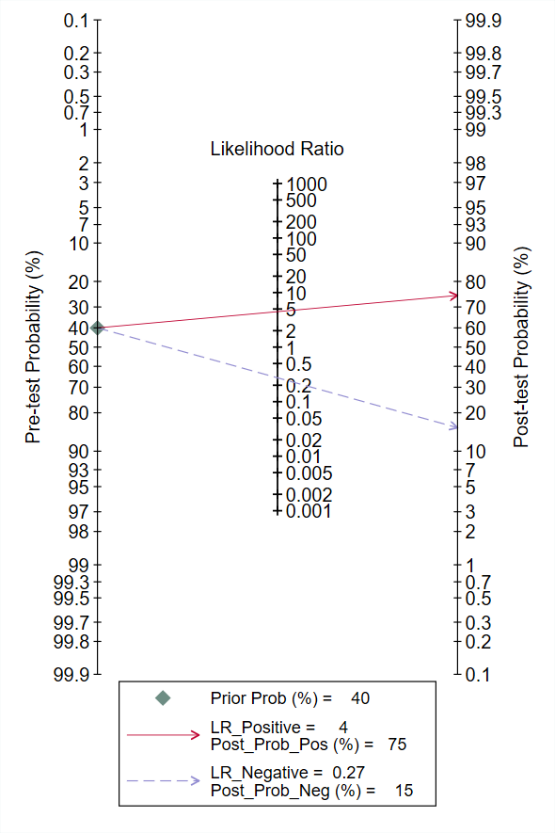


Figure S4 Fagan's nomogram from meta-analysis assessing all machine learning models for VETC diagnosis in the training se


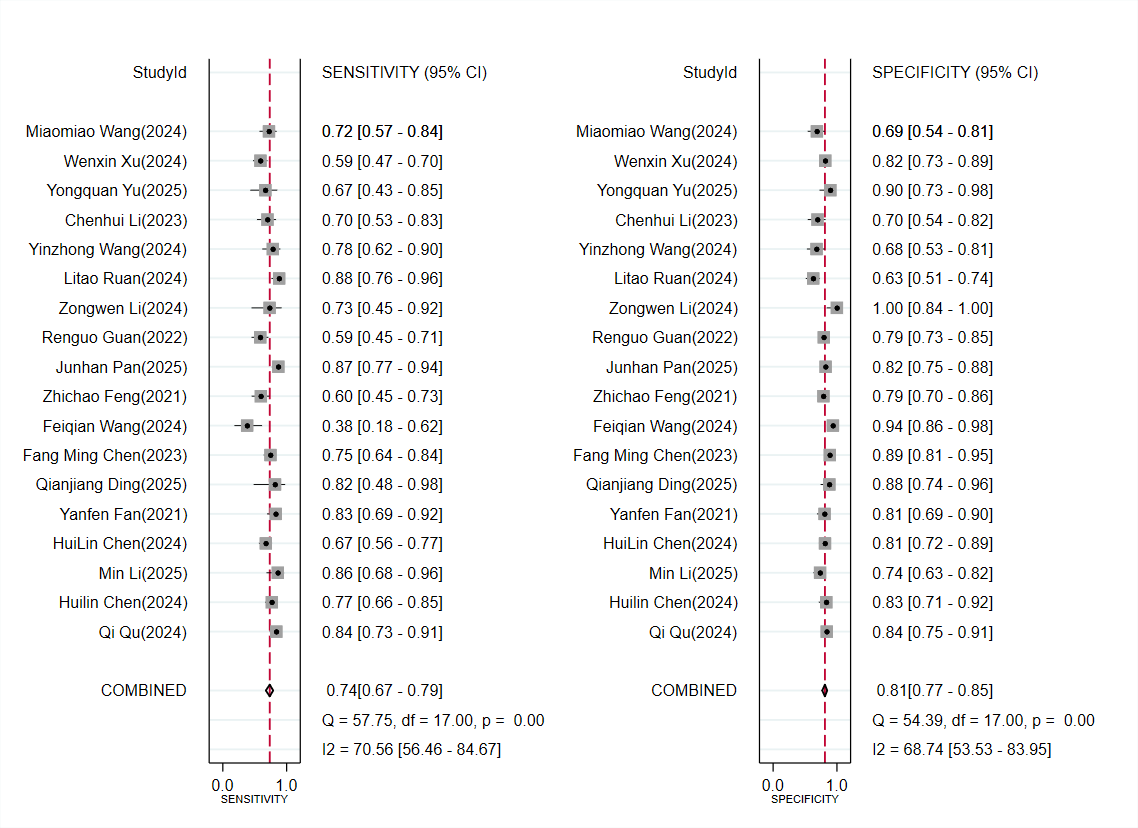
Figure S5 Forest plot from meta-analysis assessing the sensitivity and specificity of non-radiomic machine learning models for VETC diagnosis in the training set


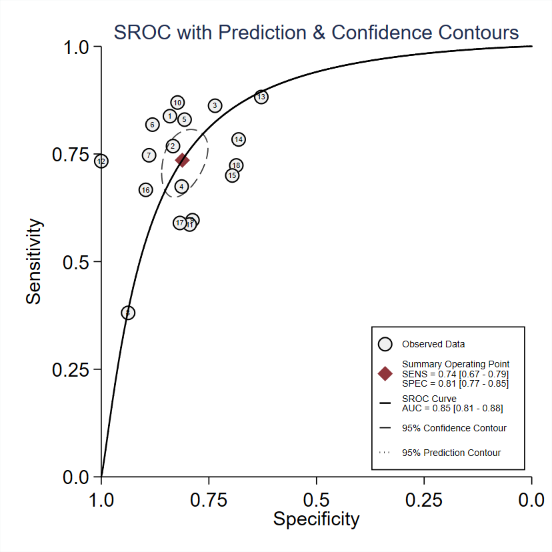


Figure S6 SROC curve from meta-analysis evaluating non-radiomic machine learning models in VETC diagnosis in the training set


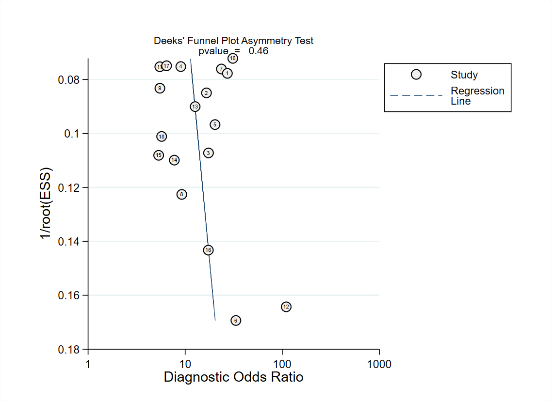


Figure S7 Deeks' funnel plot from meta-analysis examining non-radiomic machine learning models for VETC diagnosis in the training set


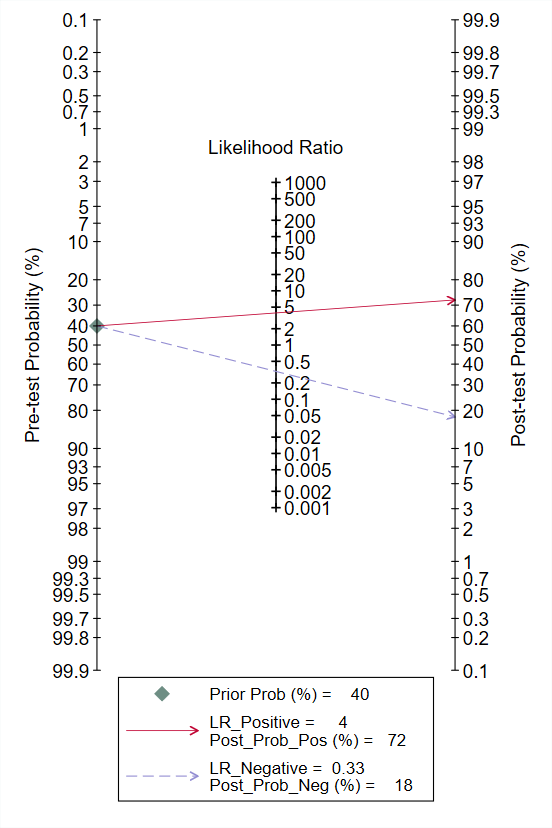


Figure S8 Fagan's nomogram from meta-analysis assessing non-radiomic machine learning models for VETC diagnosis in the training set


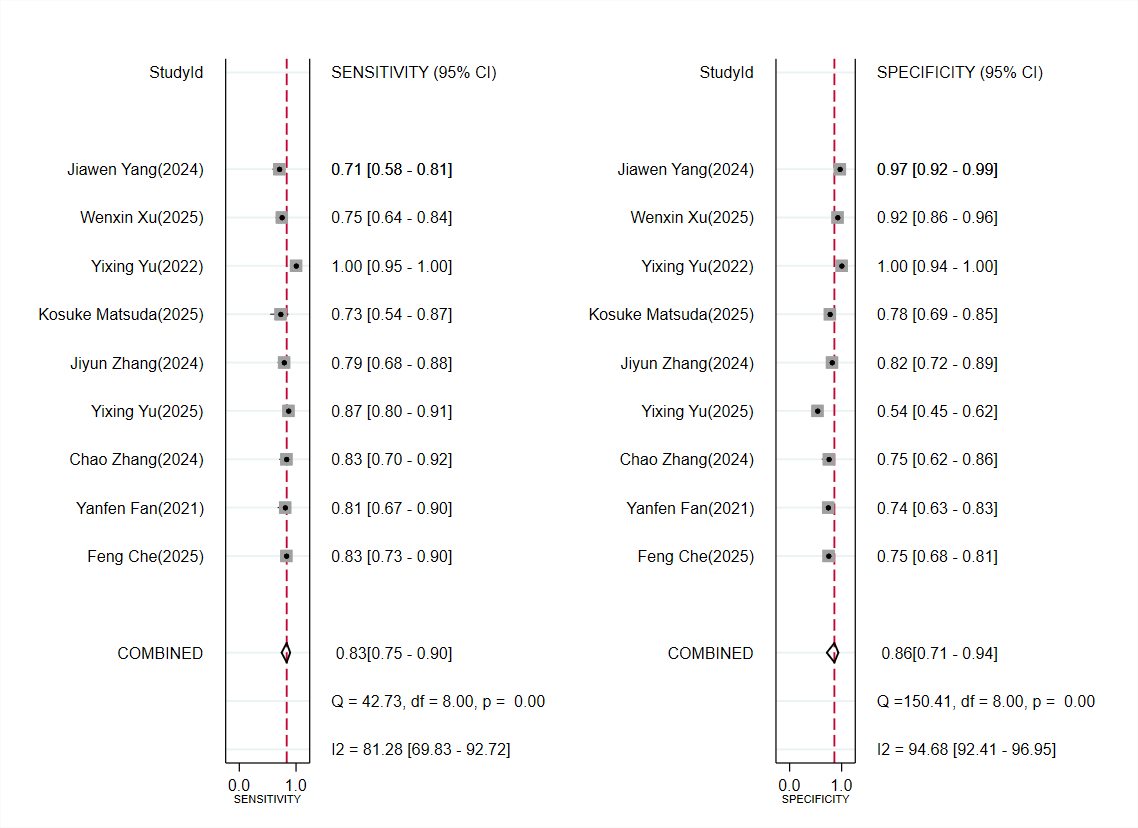


Figure S9 Forest plot from meta-analysis assessing the sensitivity and specificity of radiomic machine learning models for VETC diagnosis in the training set


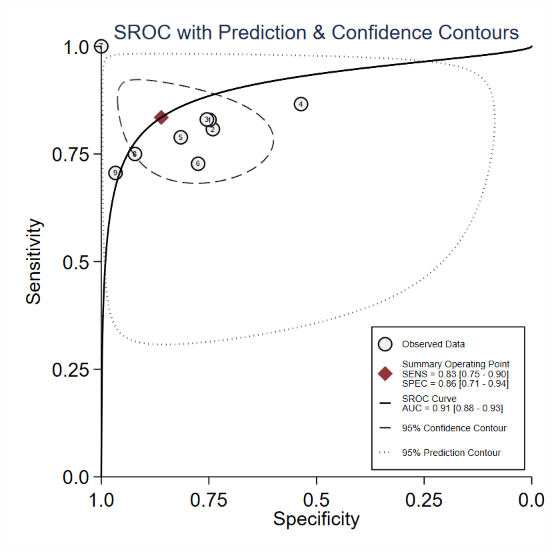


Figure S10 SROC curve from meta-analysis evaluating radiomic machine learning models in VETC diagnosis in the training set


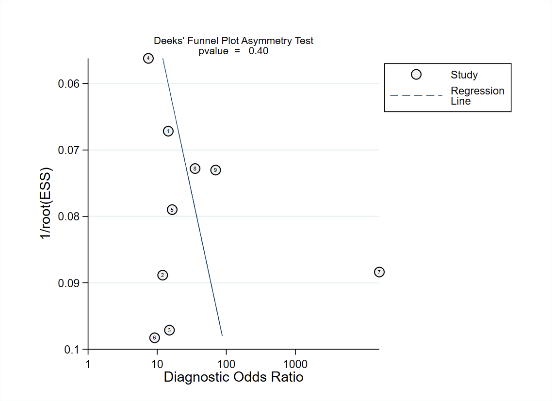


Figure S11 Deeks' funnel plot from meta-analysis examining radiomic machine learning models for VETC diagnosis in the training set


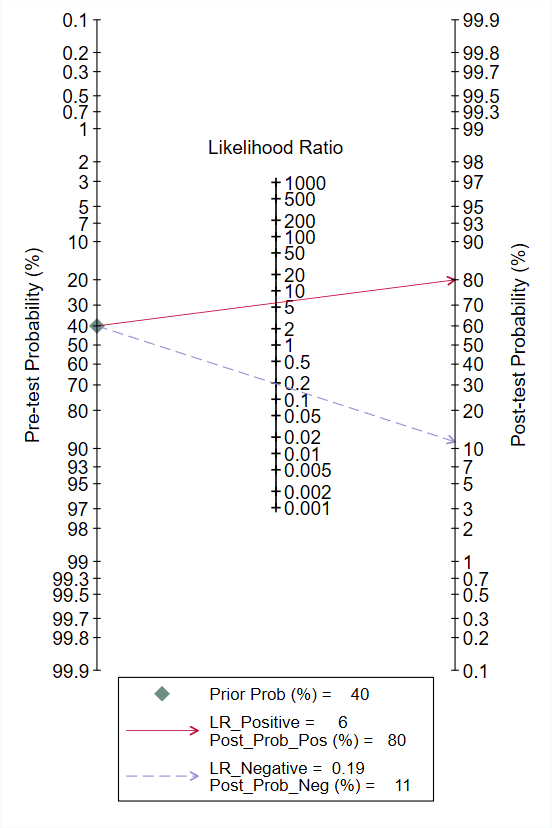


Figure S12 Fagan's nomogram from meta-analysis assessing radiomic machine learning models for VETC diagnosis in the training set


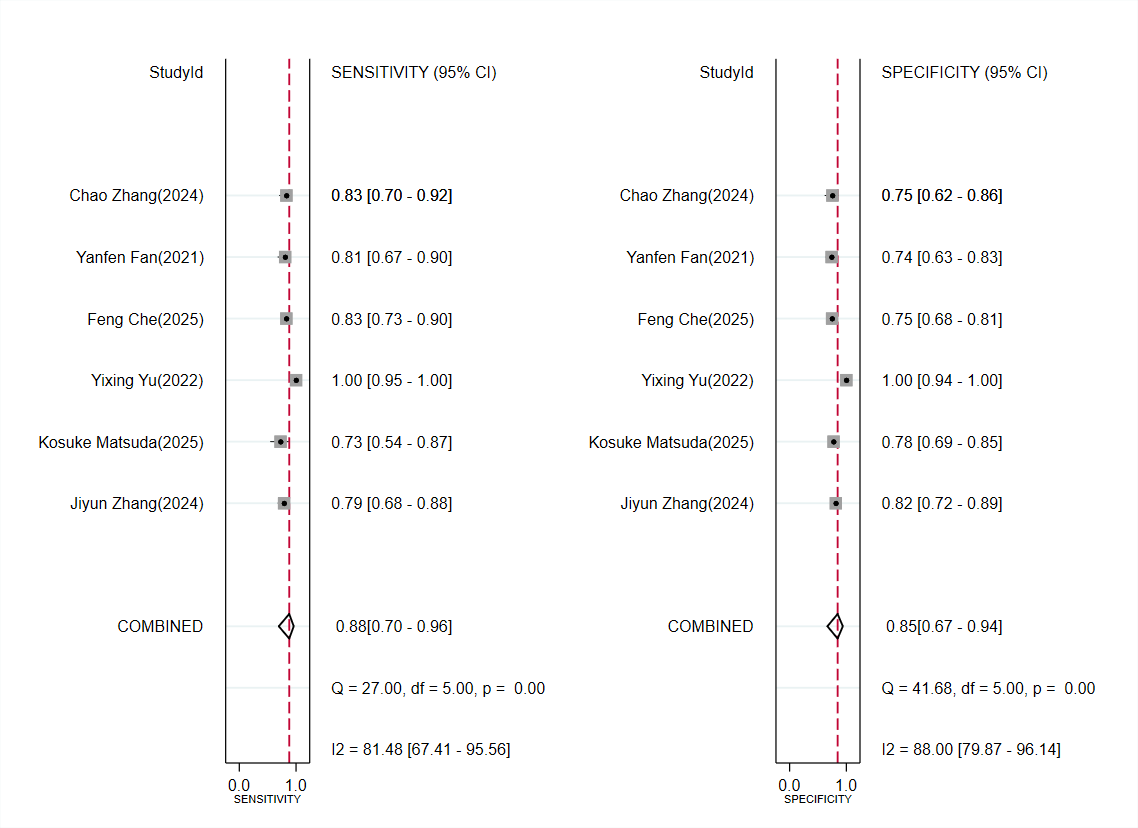


Figure S13 Forest plot from meta-analysis assessing the sensitivity and specificity of traditional machine learning models using radiomics features for VETC diagnosis in the training set


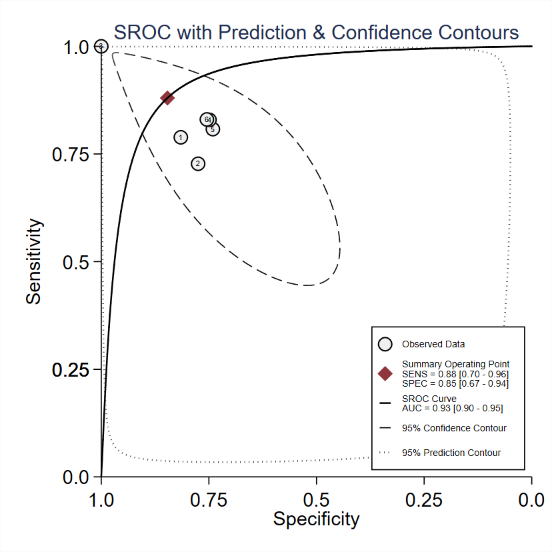


Figure S14 SROC curve from meta-analysis evaluating traditional machine learning models with radiomics features in VETC diagnosis in the training set


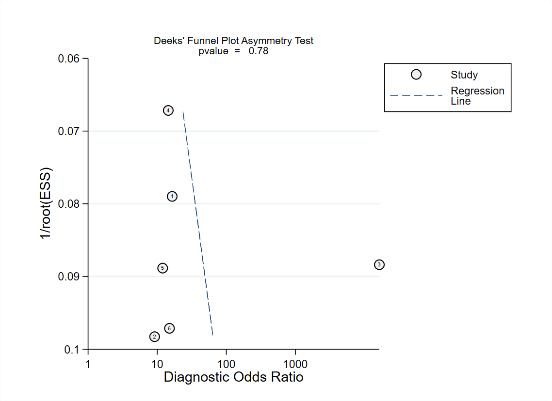


Figure S15 Deeks' funnel plot from meta-analysis examining traditional machine learning models using radiomics features for VETC diagnosis in the training set


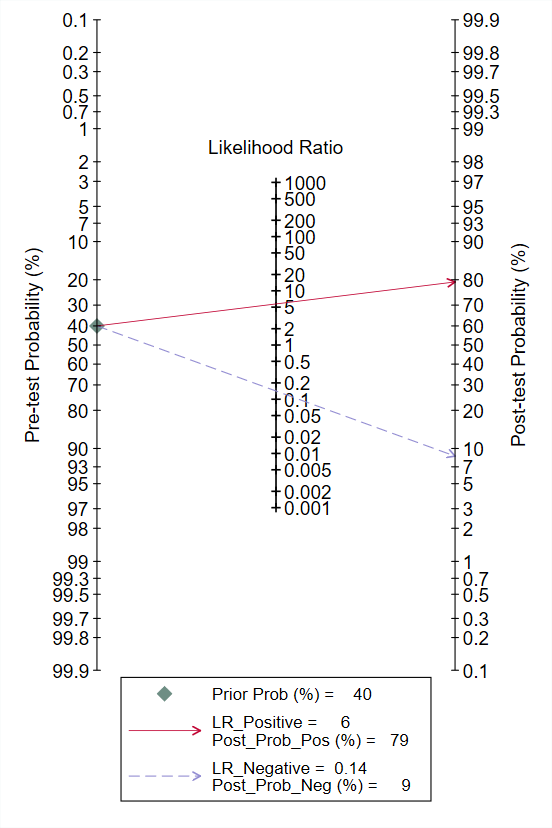


Figure S16 Fagan's nomogram from meta-analysis assessing traditional machine learning models with radiomics features for VETC diagnosis in the training set


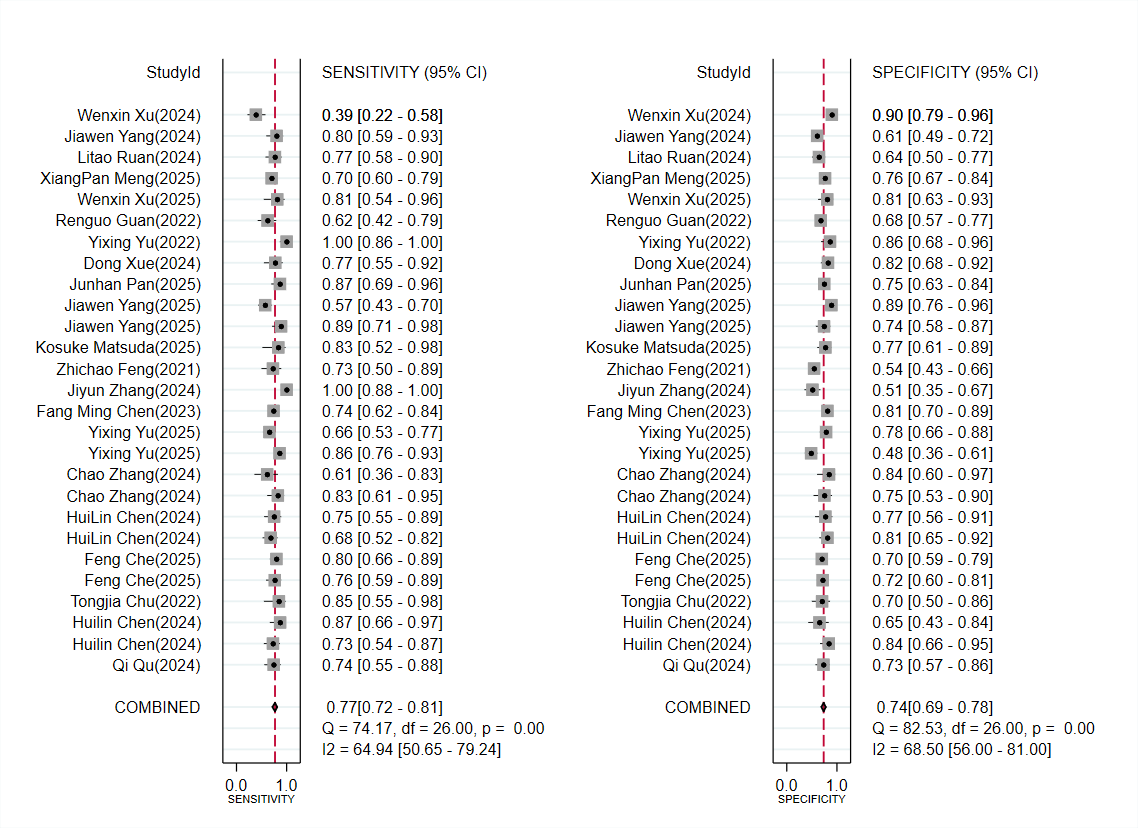


Figure S17 Forest plot from meta-analysis assessing the sensitivity and specificity of all machine learning models for VETC diagnosis in the validation set


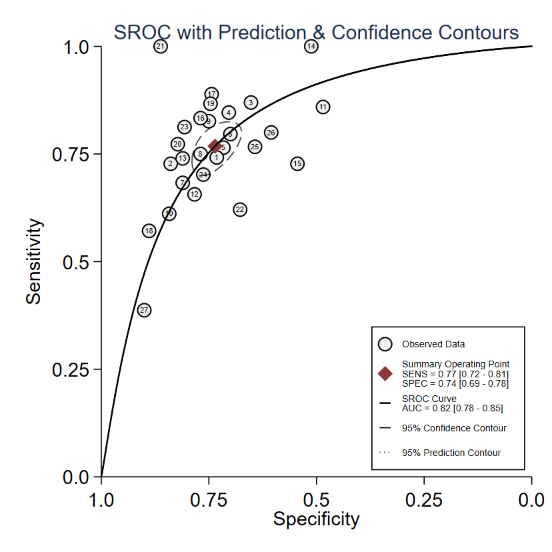


Figure S18 SROC curve from meta-analysis evaluating all machine learning models in VETC diagnosis in the validation set


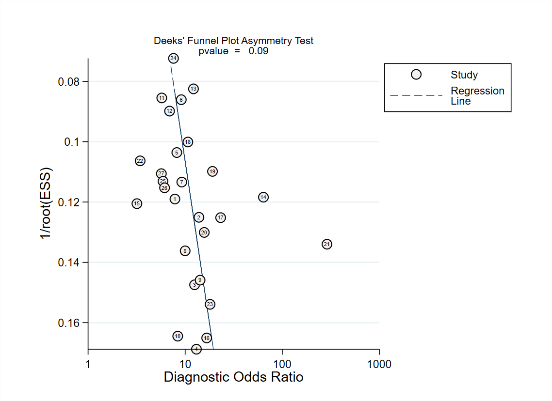


Figure S19 Deeks' funnel plot from meta-analysis examining all machine learning models for VETC diagnosis in the validation set


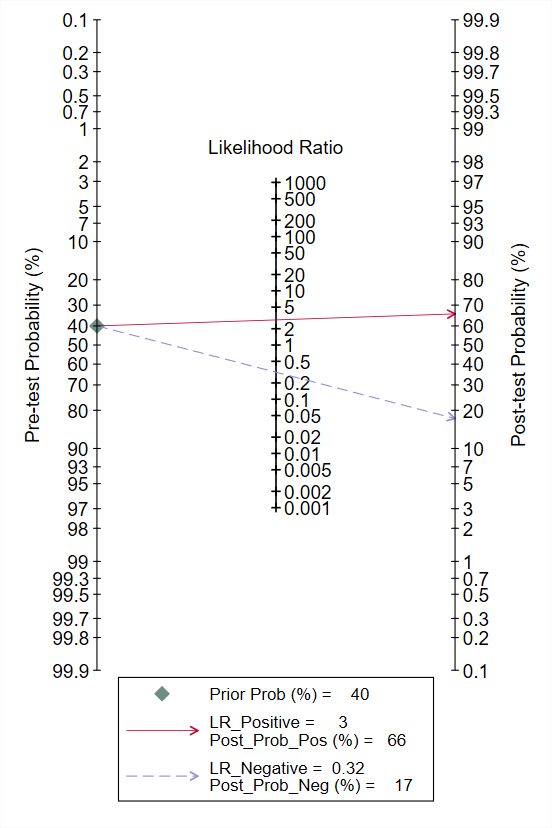


Figure S20 Fagan's nomogram from meta-analysis assessing all machine learning models for VETC diagnosis in the validation set


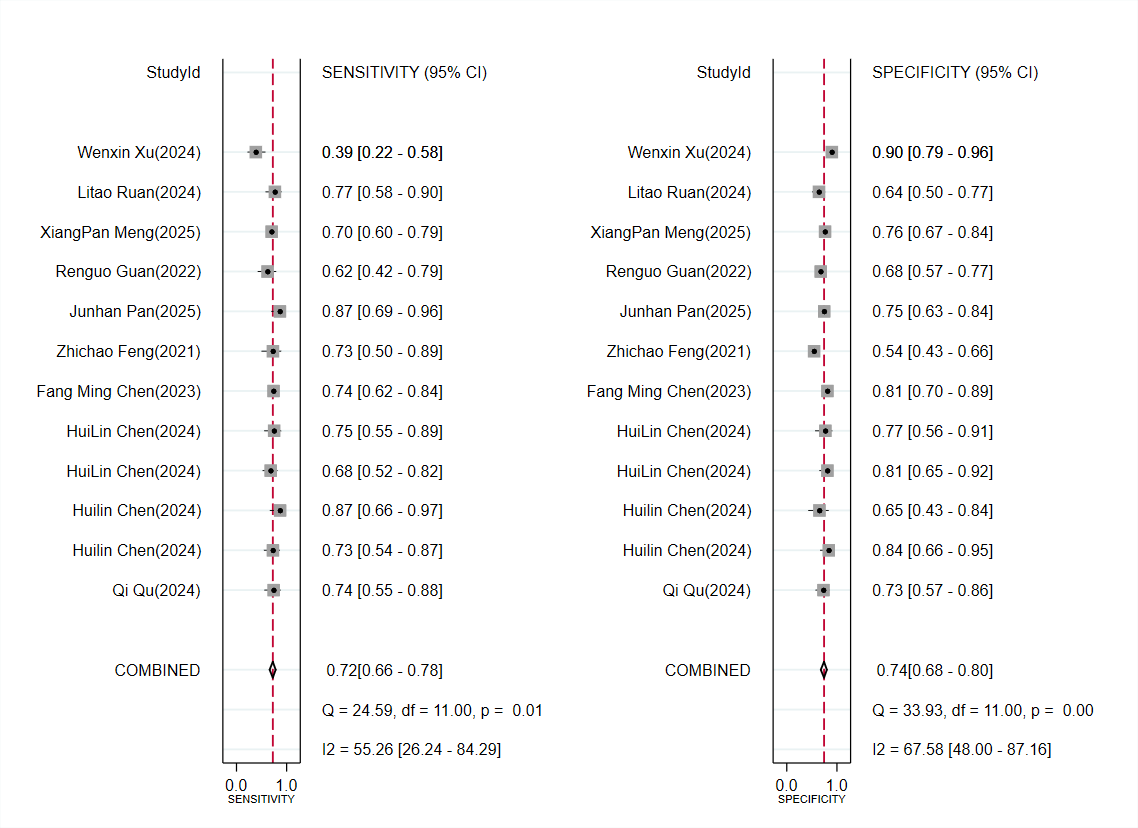


Figure S21 Forest plot from meta-analysis assessing the sensitivity and specificity of non-radiomic machine learning models for VETC diagnosis in the validation set


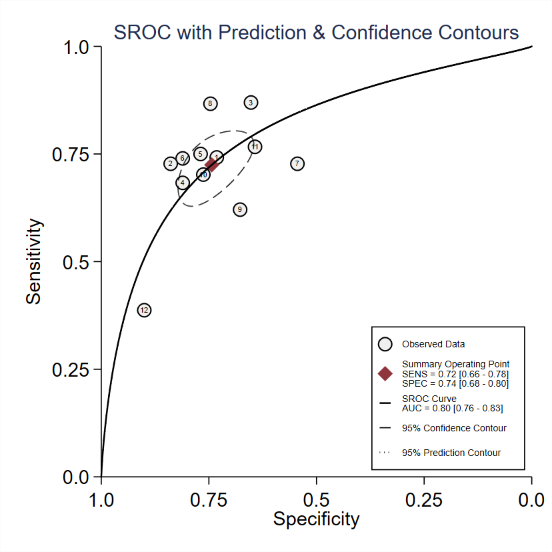


Figure S22 SROC curve from meta-analysis evaluating non-radiomic machine learning models in VETC diagnosis in the validation set


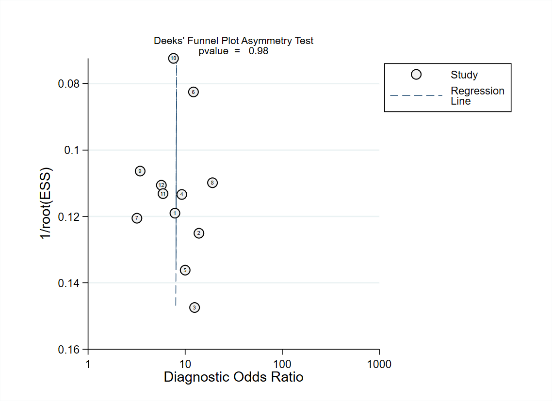


Figure S23 Deeks' funnel plot from meta-analysis examining non-radiomic machine learning models for VETC diagnosis in the validation set


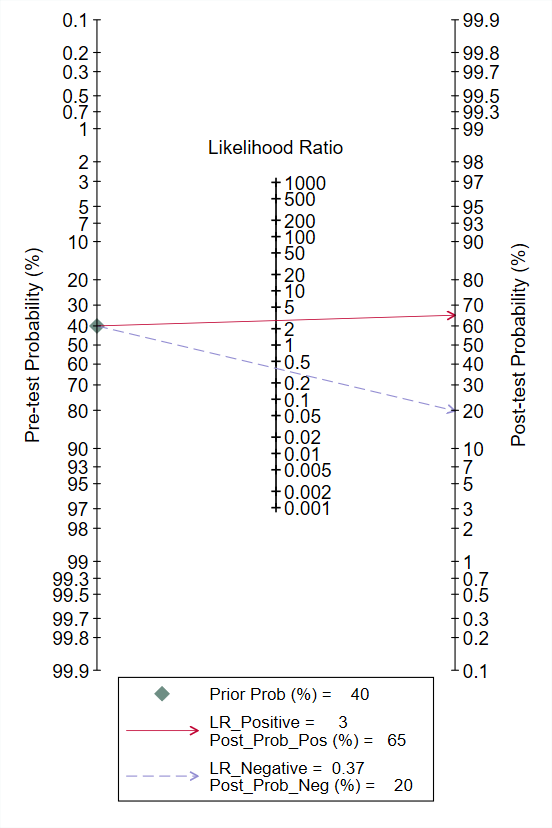


Figure S24 Fagan's nomogram from meta-analysis assessing non-radiomic machine learning modelsfor VETC diagnosis in the validation set


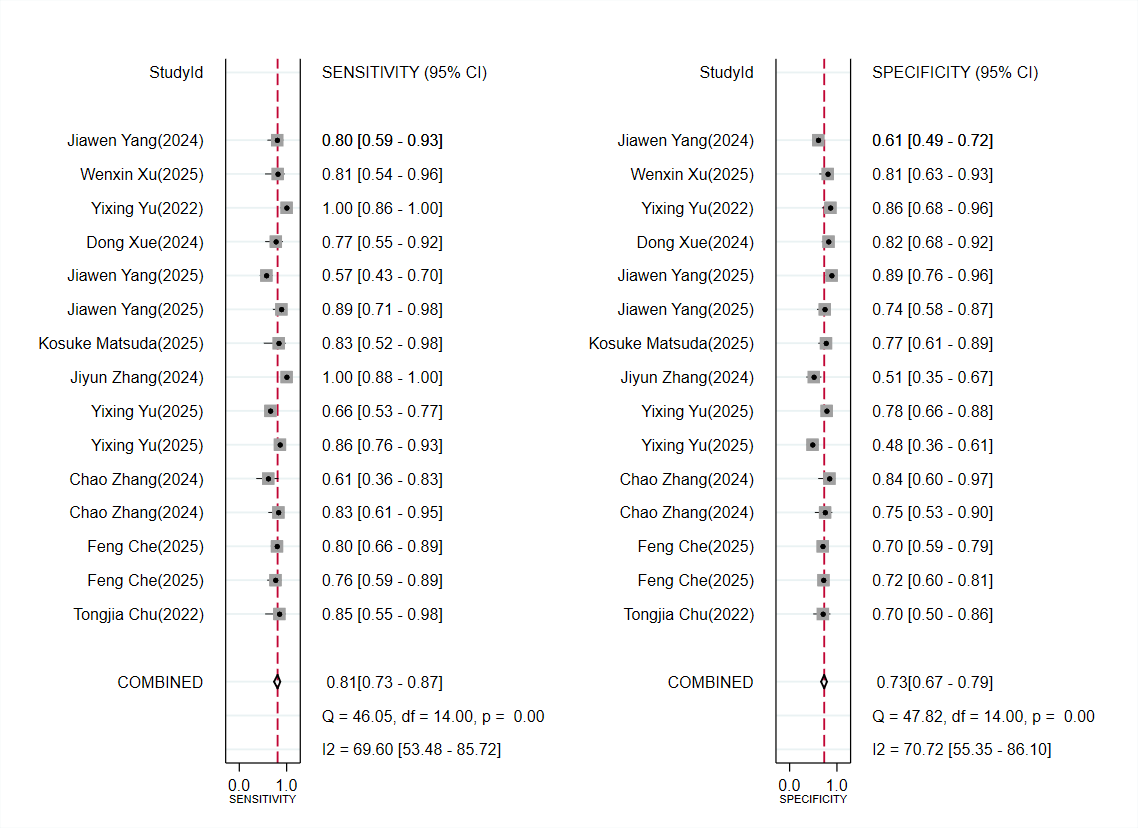


Figure S25 Forest plot from meta-analysis assessing the sensitivity and specificity of radiomic machine learning models for VETC diagnosis in the validation set


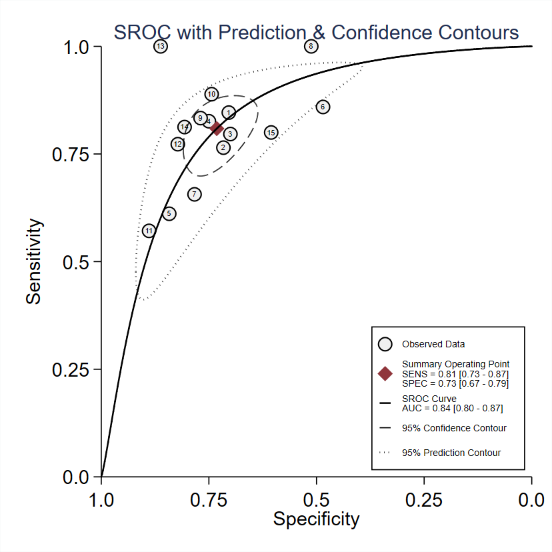


Figure S26 SROC curve from meta-analysis evaluating radiomic machine learning models in VETC diagnosis in the validation set


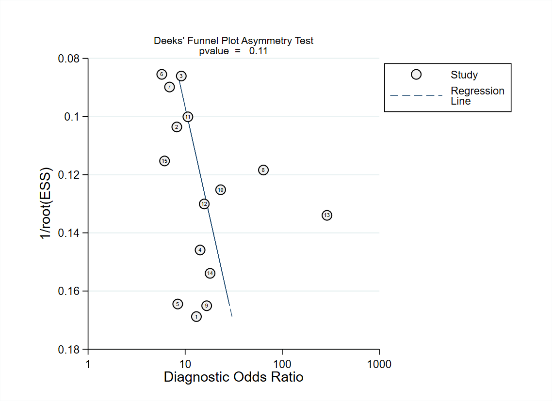


Figure S27 Deeks' funnel plot from meta-analysis examining radiomic machine learning models for VETC diagnosis in the validation set


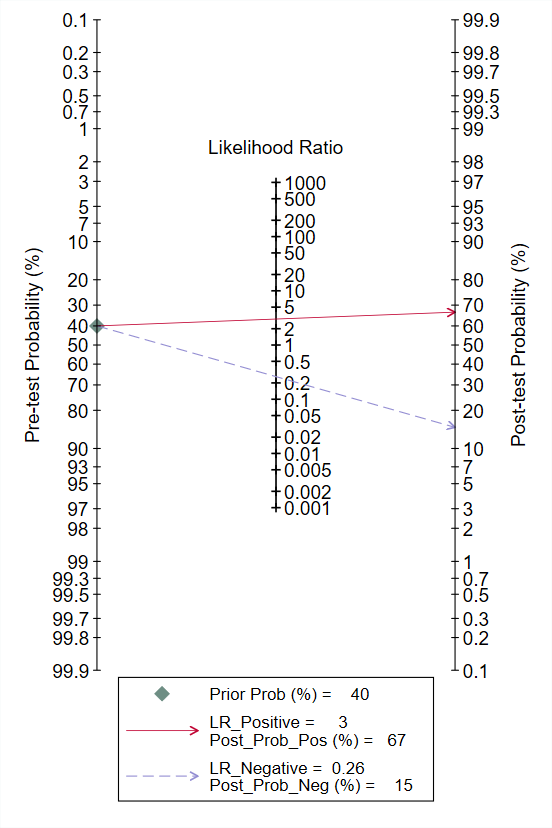


Figure S28 Fagan's nomogram from meta-analysis assessing radiomic machine learning models for VETC diagnosis in the validation set


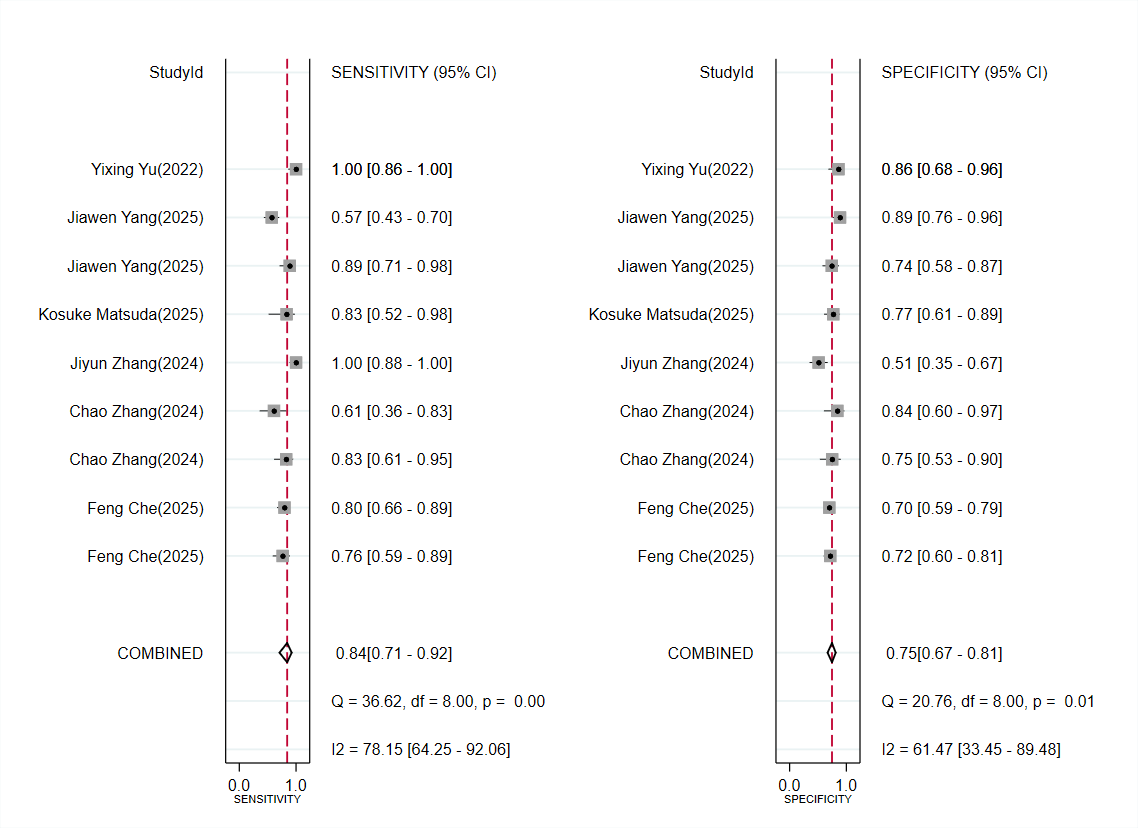


Figure S29 Forest plot from meta-analysis assessing the sensitivity and specificity of traditional machine learning models using radiomics features for VETC diagnosis in the validation set


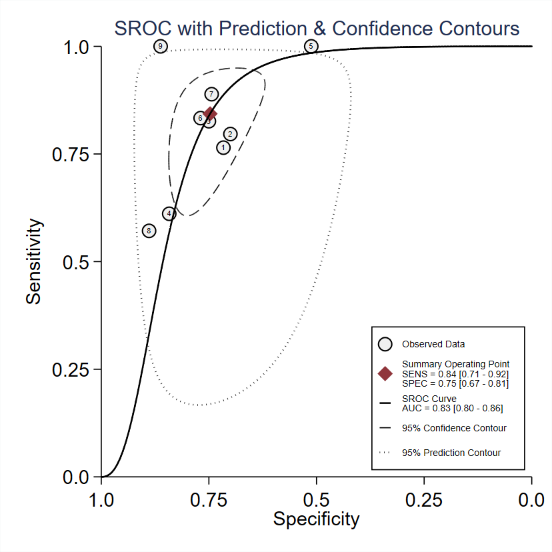


Figure S30 SROC curve from meta-analysis evaluating traditional machine learning models with radiomics features in VETC diagnosis in the validation set


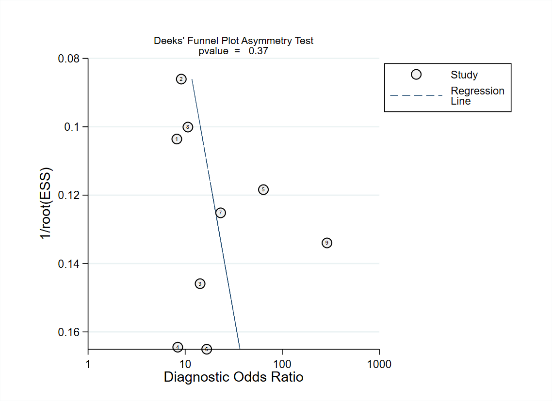


Figure S31 Deeks' funnel plot from meta-analysis examining traditional machine learning models using radiomics features for VETC diagnosis in the validation set


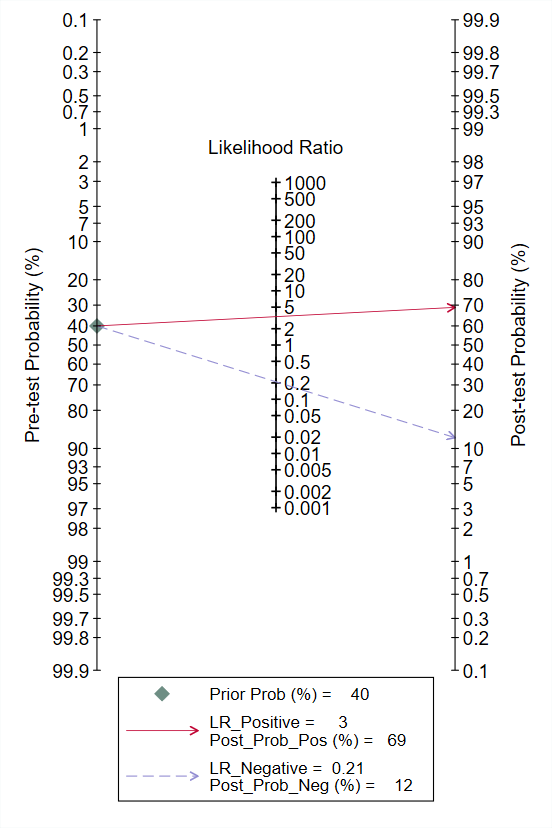


Figure S32 Fagan's nomogram from meta-analysis assessing traditional machine learning models with radiomics features for VETC diagnosis in the validation set


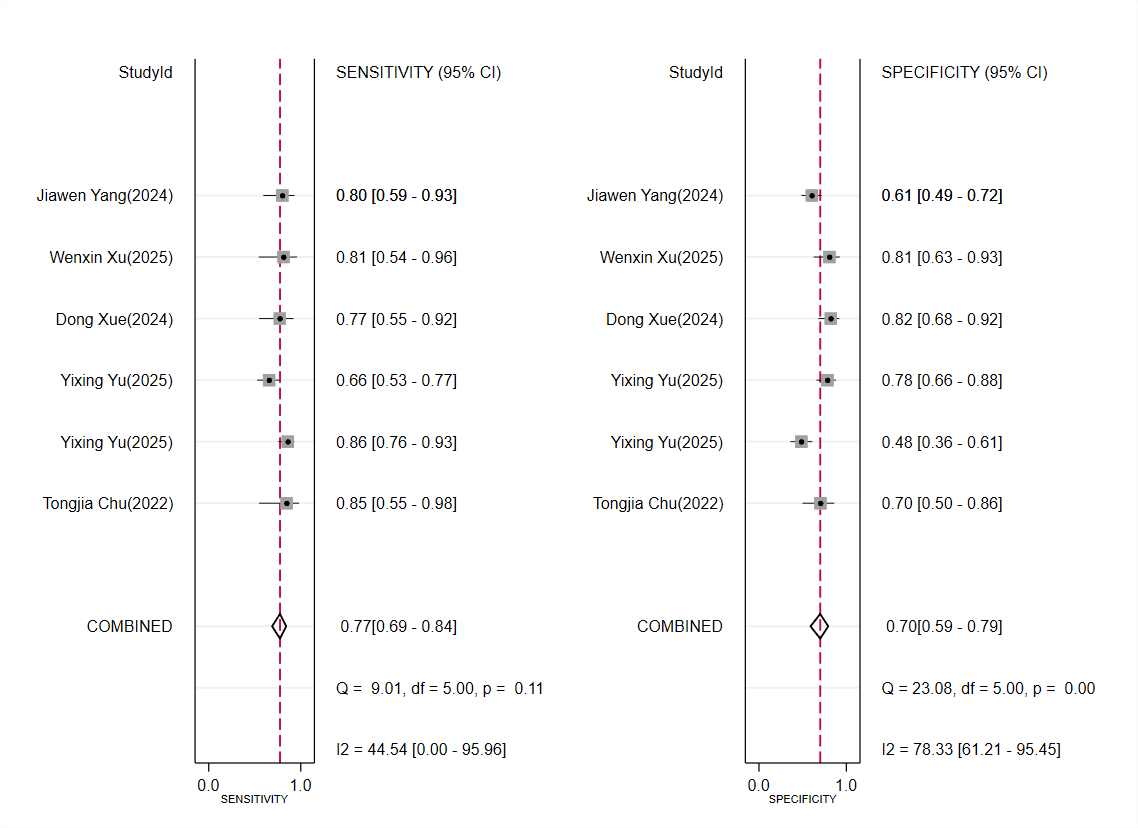


Figure S33 Forest plot from meta-analysis assessing the sensitivity and specificity of deep learning model using radiomics features for VETC diagnosis in the validation set


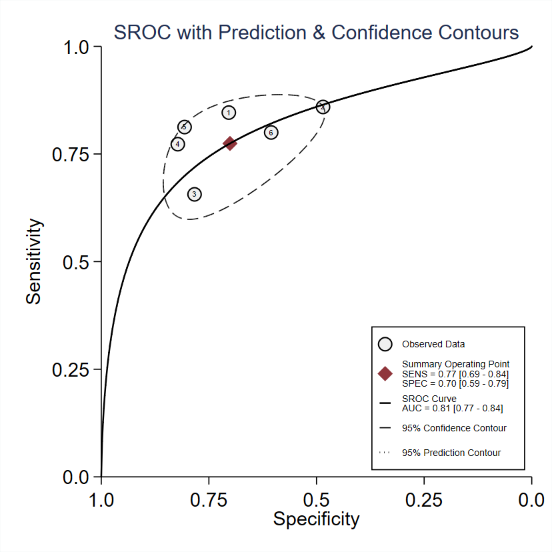


Figure S34 SROC curve from meta-analysis evaluating deep learning model with radiomics features in VETC diagnosis in the validation set


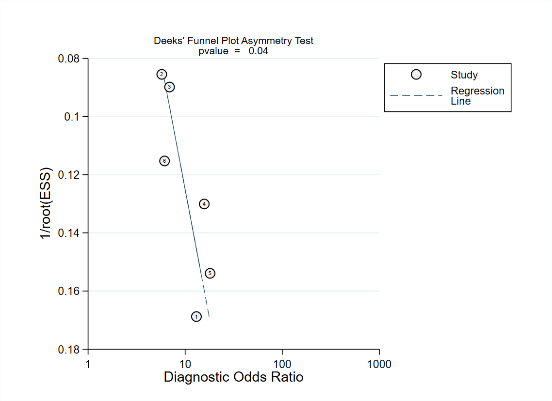


Figure S35 Deeks' funnel plot from meta-analysis examining deep learning model using radiomics features for VETC diagnosis in the validation set


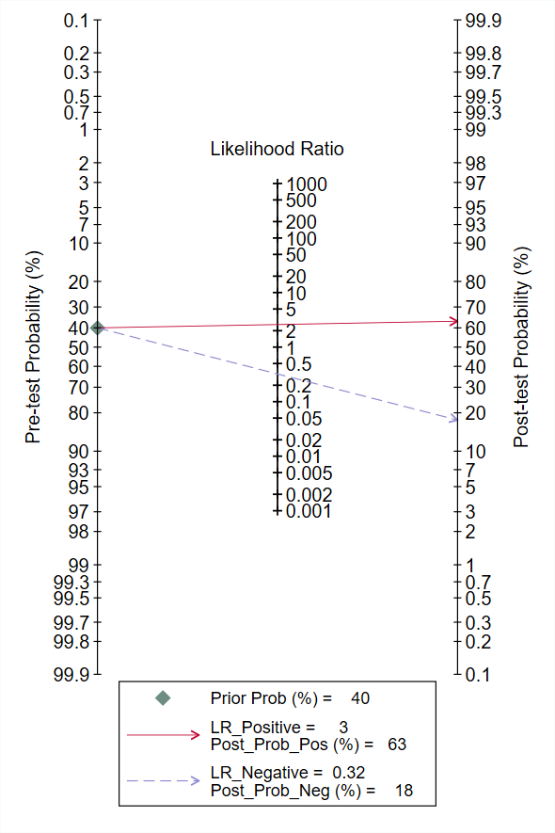


Figure S36 Fagan's nomogram from meta-analysis assessing deep learning model with radiomics features for VETC diagnosis in the validation set
